# *Sufu*- and *Spop*-mediated downregulation of Hedgehog signaling promotes beta cell differentiation through organ-specific niche signals

**DOI:** 10.1038/s41467-019-12624-5

**Published:** 2019-10-11

**Authors:** Theodora Yung, Frankie Poon, Minggao Liang, Sabrina Coquenlorge, Emily C. McGaugh, Chi-chung Hui, Michael D. Wilson, M. Cristina Nostro, Tae-Hee Kim

**Affiliations:** 1 Program in Developmental & Stem Cell Biology, The Hospital for Sick Children, Toronto, Ontario M5G 0A4 Canada; 2 Department of Molecular Genetics, University of Toronto, Toronto, Ontario M5S 1A8 Canada; 3 McEwen Stem Cell Institute, University Health Network, Toronto, Ontario M5G 2C4 Canada; 4 Department of Physiology, University of Toronto, Toronto, Ontario M5S 1A8 Canada; 5 Program in Genetics & Genome Biology, The Hospital for Sick Children, Toronto, Ontario M5G 0A4 Canada; 6 Heart & Stroke Richard Lewar Centre of Excellence in Cardiovascular Research, Toronto, Canada

**Keywords:** Differentiation, Organogenesis, Stem cells, Stem-cell niche

## Abstract

Human embryonic stem cell-derived beta cells offer a promising cell-based therapy for diabetes. However, efficient stem cell to beta cell differentiation has proven difficult, possibly due to the lack of cross-talk with the appropriate mesenchymal niche. To define organ-specific niche signals, we isolated pancreatic and gastrointestinal stromal cells, and analyzed their gene expression during development. Our genetic studies reveal the importance of tightly regulated Hedgehog signaling in the pancreatic mesenchyme: inactivation of mesenchymal signaling leads to annular pancreas, whereas stroma-specific activation of signaling via loss of Hedgehog regulators, *Sufu* and *Spop*, impairs pancreatic growth and beta cell genesis. Genetic rescue and transcriptome analyses show that these *Sufu* and *Spop* knockout defects occur through *Gli2*-mediated activation of gastrointestinal stromal signals such as Wnt ligands. Importantly, inhibition of Wnt signaling in organoid and human stem cell cultures significantly promotes insulin-producing cell generation, altogether revealing the requirement for organ-specific regulation of stromal niche signals.

## Abstract (web summary)

Dynamic mesenchyme derived signals are known to direct proper organ formation and cell specification in vivo. Here the authors show in mice that mesenchyme derived Hedgehog and Wnt instruct the formation of the pancreas and beta cells, and that Wnt inhibition promotes beta cell formation from human pluripotent cells.

## Introduction

Dynamic mesenchymal niche signals and diverse signaling pathways govern the formation of insulin-secreting beta cells in the pancreas in vivo. Recent advances in the in vitro differentiation of human embryonic stem cells (hESCs) into beta cells has demonstrated the increasingly promising application of cell-based therapies for diabetes^1–3^. However, the differentiation of these progenitors into insulin-producing cells remains inefficient and presents a significant challenge in the efficacy and economics of stem-cell based therapies.

The critical reliance of pancreatic epithelium on its surrounding mesenchyme is well-established: loss of mesenchyme at any stage of murine pancreatogenesis arrests or impairs development, while growth is facilitated by co-culture with its organ-matched mesenchyme, and culture with non-matched mesenchyme can influence differentiation^4–7^. Microarray analysis of early embryonic (E)11.5 pancreatic, stomach, and intestinal mesenchyme has allowed for the identification of factors beneficial to beta cell differentiation^8^. Notably, this analysis showed overwhelmingly similar expression patterns among these organs, implying that organ-specific niches have yet to be established at this stage^8^. Later investigations have been limited due to the rapid branching of epithelium into mesenchyme after E11.5, making manual separation nearly impossible. Dramatic epithelial transformation of digestive organs occurs during mid-development, which includes pancreatic lineage commitment and intestinal villification^9,10^. Expression analyses between the pancreatic epithelium and mesenchyme identified hundreds of tissue-specific genes at E12.5, suggesting that significant gene expression changes occur in mid-development^11^. Thus, organ-specific niches may be established during this critical period to guide each organ’s epithelial differentiation, and investigating these mechanisms could prove informative to beta cell generation. Notably, further investigation of digestive organ mesenchyme was recently made possible by the development of a *Bapx1*^*Cre*^ mouse allele to label pancreatic, stomach and intestinal mesenchyme^6,12,13^.

Key pathways play critical roles during pancreatic development. In contrast to its inductive role in most organ development, Hedgehog (Hh) signaling inhibits pancreatic organogenesis, with ectopic activation in either the epithelium or mesenchyme inducing hypoplasia and beta cell impairment^14,15^. Despite these inhibitory roles, Hh reporter mice display active expression in both pancreatic epithelium and mesenchyme, suggesting the presence of low-level signaling rather than a complete exclusion^16^. Interestingly, epithelial-specific Hh signaling inhibition does not recapitulate the pancreatic defects seen with global inhibition, implying a mesenchyme-specific requirement for Hh signaling not yet explored^16,17^.

Hh signaling is mediated by key regulators that act on its downstream GLI transcription factors (TFs). Suppressor of Fused (SUFU) sequesters GLI TFs in the cytoplasm, while the more recently discovered Speckle-type POZ protein (SPOP,  also known as PCIF1) targets them for proteasomal degradation^18,19^. Recently, SPOP was demonstrated to have the ability to promote and inhibit Hh signaling in the mouse skeleton and neural tube, respectively, highlighting its context-specific roles^20,21^. In the murine pancreas, SPOP has been suggested to negatively regulate beta cell gene expression, but the role of SPOP in the context of pancreatic Hh signaling is unknown^22^.

In addition to Hh, Wnt signaling must also be suppressed for pancreatic development^23^. While genetic knockout of Wnt signaling produces either exocrine or endocrine defects depending on the manipulation method^24,25^, its ectopic activation impairs pancreatic growth and specification, suggesting the requirement for tightly controlled Wnt signaling^6,26,27^. However, the role of Wnt signaling in beta cell differentiation and its relationship with Hh signaling is unclear.

Here we employ *Bapx1*^*Cre*^ reporter mice to demonstrate organ-specific mesenchymal expression patterns in the stomach, intestine, and pancreas. We utilize genetic mouse models to reveal the spatial and temporal roles of *Sufu* and *Spop* in maintaining tightly regulated, low-level Hh signaling in the pancreatic mesenchyme for proper organ size and beta cell formation. Applying our findings in organoid and human stem cell culture, we demonstrate the significance of Wnt signaling regulation in beta cell generation.

## Results

### Organ-specific niches underlie digestive organ development

**Fig. 1:**
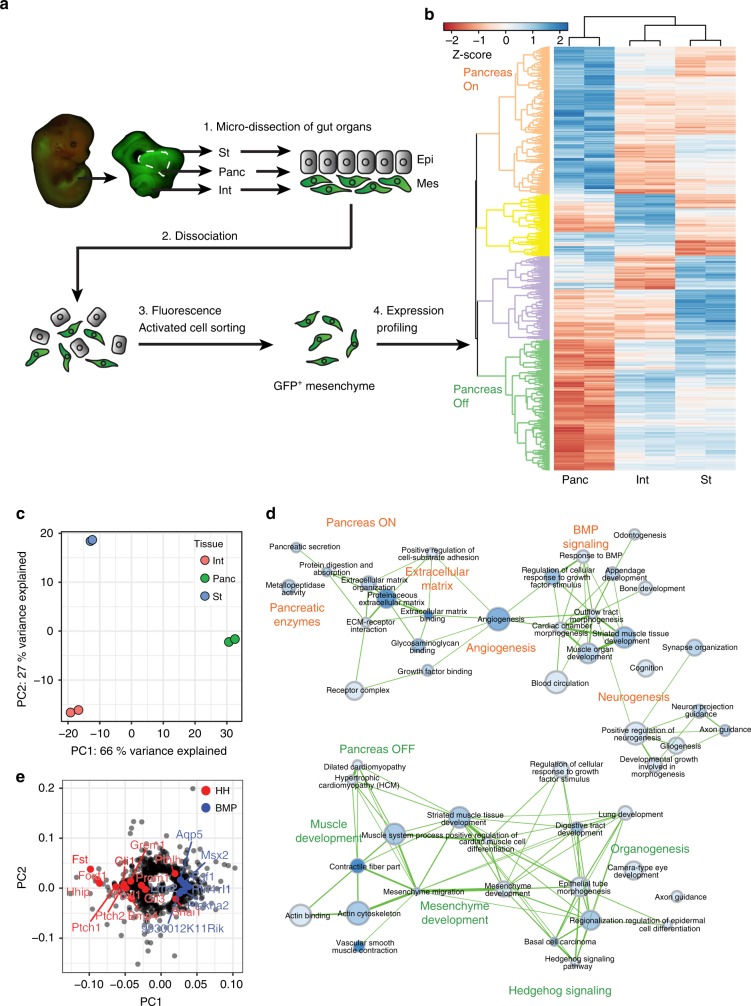
Organ-specific mesenchymal gene signatures underlie digestive organ development. **a** For organ-specific mesenchymal investigations of E13.5 gut derivatives, *Bapx1*^*Cre/+*^;*ROSA26*^*mT/mG*^ mesenchymal reporters were generated and single cell suspensions of stomach, pancreas, and intestine were prepared from each organ type. Fluorescence activated cell sorting was used to isolate GFP^+^ mesenchymal cells for RNA-sequencing analyses. **b** Unsupervised hierarchical clustering of all significantly differentially expressed genes in stomach (St), pancreatic (Panc), and intestinal (Int) mesenchyme. Plot is scaled by the Z-score of log-scaled DESeq2 normalized counts, with increasing values (from red to blue) indicating relative enrichment. **c** Principal component analysis showing separation of stomach, intestinal, and pancreatic mesenchymal transcriptomes by tissue of origin. **d** GO term enrichment analyses of genes differentially regulated in the pancreatic mesenchyme compared to the stomach and intestinal mesenchyme (*p* < 0.01; hypergeometric test with Bonferroni correction). Nodes are shaded based on enrichment *p*-value and sized based on the number of genes in a given set. Edges are weighted by the number of genes shared between terms (minimum overlap of 0.2). **e** Loadings on PC1 identify Hedgehog (HH) and BMP pathway members and targets as differentiating the pancreas from stomach and intestinal mesenchyme

To identify organ-specific niche factors and define mesenchymal-epithelial interactions in digestive organ development, we generated E13.5 *Bapx1*^*Cre/+*^;*ROSA26*^*mT/mG*^ reporter embryos. This reporter system allows for the fluorescence-activated cell sorting and transcriptomic analysis of GFP^+^ *Bapx1*-expressing pancreatic, stomach and intestinal mesenchymal cells^6,13^ (Fig. 1a, Supplementary Fig. 1A). Differential expression analyses between tissue pairs identified a total of 995 genes that were significantly differentially expressed (DE) in at least one comparison, demonstrating the existence of organ-specific niches (Supplementary Data 1-3). Unsupervised hierarchical clustering of DE genes identified four groups, with the largest clusters containing genes selectively downregulated (Pancreas OFF) or upregulated in the pancreas (Pancreas ON) (Fig. 1b). Consistently, principal component analysis (PCA) showed clear separation of mesenchymal cells based on their tissue of origin (Fig. 1c). Pancreatic mesenchymal cells separated furthest from other organs along PC1, suggesting that they exhibit the most distinct signature among the organs profiled (Fig. 1c).

To better understand the regulatory networks maintaining tissue-specific niches, we inferred regulatory interactions between expressed TFs and DE genes using an approach that integrates motif-based TF-target gene mapping and co-expression data^28^. We mapped TFs to target genes via promoter motif discovery and utilized co-expression data from an independent compendium of mouse tissues^29^. We considered any gene annotated as a TF with a known DNA-binding motif that was expressed in at least one organ type profiled^30^. To identify candidate niche regulators, we examined individual TFs for enrichment of regulatory edges to genes in a given cluster, identifying organ-specific regulators (Supplementary Fig. 1B-D). For example, TFs enriched for interactions with Pancreas ON genes include regulators of pancreas gene expression such as *Irf6* and *Usf1*^31,32^, as well as previously unreported factors, *Foxk1* and *Six5*.

To define the signaling mechanisms underlying organ-specific niches, we performed Gene Ontology (GO) enrichment analysis on each cluster. Top GO terms for the Pancreas ON cluster include neurogenesis and extracellular matrix interactions, as well as BMP signaling, which indeed has been demonstrated to be active in the chick pancreatic mesenchyme^33^. The Pancreas OFF cluster was enriched for terms related to muscle development and Hh signaling (Fig. 1d). Consistent with these findings, Hh targets (*Gli1, Ptch1, Foxf1*) were highly enriched in the Pancreas OFF cluster (*p* < 8.72E-14; hypergeometric test with Bonferroni correction), while the Pancreas ON cluster showed an enrichment for BMP target genes (*p* < 1.57E-3; hypergeometric test with Bonferroni correction). Likewise, visualization of PCA loadings showed that the majority of Hh targets have negative loading values for PC1, while BMP targets have positive loading values, suggesting Hh- and BMP−mediated regulation of gastrointestinal and pancreatic mesenchymal identity (Fig. 1e). Together, our findings indicate that differential Hh and BMP signaling may drive distinct digestive organ-specific niche signals.

### Mesenchymal *Sufu* is required for pancreatic development

While our data suggest the down-regulation of pancreatic mesenchymal Hh signaling, the expression of *Ptch1* in the pancreatic epithelium and mesenchyme of Hh reporter mice indicates the existence of active signaling^16^. Together this suggests the presence of tightly regulated, low-level Hh signaling in the pancreas. Intracellular Hh regulators, SUFU and SPOP, control the final balance of GLI effectors to modulate diverse physiological activities throughout the body^18^. We therefore examined their roles in pancreatic development. To assess the spatial and temporal expression of *Sufu* and *Spop* in the developing pancreas, we performed single molecule fluorescent *in situ* hybridization (smFISH) using our GFP-mesenchymal reporter (*Bapx1*^*Cre/+*^;*ROSA26*^*mT/mG*^). Robust expression of *Sufu* and *Spop* can be detected in both the GFP^-^ epithelium and GFP^+^ mesenchyme during pancreatic specification and continuing throughout embryogenesis (Supplementary Fig. 2A, C, F-H, K-M). Co-staining with smooth muscle actin (SMA, to mark blood vessels) and desmin (DES, to mark mesenchyme) did not identify any sub-localization of *Sufu* and *Spop* (Supplementary Fig. 2I, J, N, O).

**Fig. 2:**
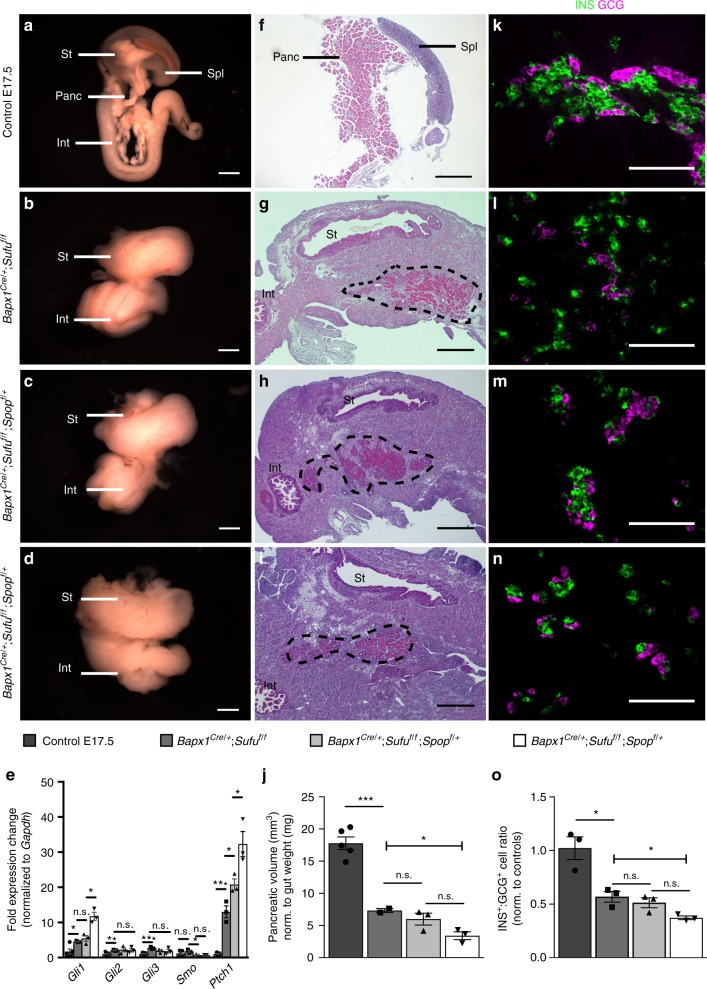
Loss of mesenchymal *Sufu* and *Spop* impairs pancreatic growth and differentiation. **a** E17.5 control embryonic gut displaying stomach (St), spleen (Spl), pancreas (Panc), and proximal intestine (Int). **b** Mesenchymal *Sufu* knockout (*Bapx1*^*Cre/+*^;*Sufu*^*f/f*^) exhibiting loss of external pancreas. **c** Heterozygous loss of *Spop* in *Sufu* knockout background (*Bapx1*^*Cre/+*^;*Sufu*^*f/f*^;*Spop*^*f/+*^). **d** Mesenchymal loss of both *Sufu* and *Spop* (*Bapx1*^*Cre/+*^;*Sufu*^*f/f*^;*Spop*^*f/f*^) leads to severe morphological dysregulation. **e** qPCR gene expression analysis for Hedgehog pathway members and targets in pancreatic mesenchyme upon *Sufu* and *Spop* deletion (*n* = 3 samples per genotype). **f**–**i** Histological images distinguishing highly cytoplasmic pancreatic tissue (pink eosin staining) in E17.5 control (**f**), *Bapx1*^*Cre/+*^;*Sufu*^*f/f*^ (**g**), *Bapx1*^*Cre/+*^;*Sufu*^*f/f*^;*Spop*^*f/+*^ (**h**), and *Bapx1*^*Cre/+*^;*Sufu*^*f/f*^;*Spop*^*f/f*^ (**i**) embryos (dashed line outlines pancreas). **j** Quantification of pancreatic volume (mm^3^) normalized to gut weight (mg) in *Sufu* and *Spop* mesenchymal mutants (*n* = 5 control samples, *n* = 3 samples per mutant genotype). **k**–**n** Immunostaining for INS and GCG in E17.5 control (**k**), *Bapx1*^*Cre/+*^;*Sufu*^*f/f*^ (**l**), *Bapx1*^*Cre/+*^;*Sufu*^*f/f*^;*Spop*^*f/+*^ (**m**), and *Bapx1*^*Cre/+*^;*Sufu*^*f/f*^;*Spop*^*f/f*^ (**n**) pancreata. **o** Assessment of INS^+^ to GCG^+^ cell number ratios in *Sufu* and *Spop* compound mutants, normalized to controls (*n* = 3 samples per genotype). Data are means ± SEM. n.s. denotes not significant, * denotes *p* < 0.05, ** denotes *p* < 0.005, *** denotes *p* < 0.0005 by Student’s un-paired, two tailed *t*-test. Scale bars: Whole mount; 1 mm, Histology; 500 µm, Immunofluorescence; 100 µm

To determine the necessity for down-regulated mesenchymal Hh signaling, we conditionally deleted Hh negative regulator, *Sufu*, in the pancreatic mesenchyme by generating *Bapx1*^*Cre/+*^;*Sufu*^*f/f*^ embryos (*Sufu* mesKO). *Sufu* mesKO mice died shortly after birth whereas heterozygous *Sufu* loss produced no obvious phenotypes (Supplementary Fig. 3A). At E17.5, *Sufu* mesKO guts exhibited dramatic mesenchymal hyperplasia and the striking loss of a recognizable pancreas (Fig. 2a, b). Expression of Hh target genes, *Gli1* and *Ptch1*, was significantly upregulated in mutant pancreatic mesenchyme, confirming the abnormal activation of Hh signaling in the absence of *Sufu* (Fig. 2e).

Histological staining of cross-sectioned tissue revealed the integration of cytoplasmic pancreatic-like tissue into the surrounding gut of *Sufu* mesKOs (Fig. 2f, g). However, this mutant tissue was significantly reduced in volume compared to E17.5 controls (Fig. 2j). Notably, while these mutants exhibit significant mesenchymal hyperplasia, their gut weights remain comparable to littermate controls (Supplementary Fig. 3B). As pancreatic volume was normalized to gut weight, this change was likely due to impaired epithelial growth rather than just increased mesenchyme. Immunostaining for pancreatic markers, amylase (AMY) of the exocrine compartment, and insulin (INS) of the endocrine compartment indicates proper lineage specification (Supplementary Fig. 3E, F). Further, PDX1 staining demonstrated proper localization to the hindstomach, intestine, and pancreas (Supplementary Fig. 4A, B). While *Sufu* mesKO pancreata are properly specified, they possess a significant imbalance between the two most prevalent endocrine cell-types, insulin-producing beta cells and glucagon (GCG)-producing alpha cells, with a significant drop in the ratio of INS^+^ to GCG^+^ cells (Fig. 2k, l, o). These results demonstrate the critical requirement for Hh regulator, *Sufu*, in pancreatic growth and endocrine differentiation.

The integration of pancreatic tissue into the surrounding gut-like mesenchyme of *Sufu* mesKOs suggests a change in mesenchymal identity (Fig. 2f, g). Smooth muscle markers, SMA and SM22, are normally found in the gastrointestinal mesenchyme, but are not characteristic of the pancreatic mesenchyme where they mark blood vessels (Supplementary Fig. 3I, J, L, M). However, the mesenchyme surrounding *Sufu* mesKO pancreata stains strongly for these markers, suggesting a gain of gastrointestinal mesenchymal character (Supplementary Fig. 3K, N).

### Loss of mesenchymal *Spop* exacerbates *Sufu* mutant defects

Given the requirement for Hh regulation by mesenchymal *Sufu* and the fact that multiple regulators modulate the balance of GLI to allow for context-dependent transcriptional responses, we sought to elucidate the contributions of the much lesser understood regulator, *Spop*. To address this, we crossed *Bapx1*^*Cre/+*^ mice to mice harboring a *Spop* floxed allele to generate *Bapx1*^*Cre/+*^;*Spop*^*f/f*^ progeny (*Spop* mesKO). *Spop* mesKO mice survived into adulthood and embryos appeared phenotypically normal with an external pancreas, comparable organ size, and predominantly INS^+^ endocrine makeup (Supplementary Fig. 5A-C).

SUFU and SPOP have been shown to compete for GLI binding in vitro, suggesting potential redundancy^18,19^. To address this, we crossed *Bapx1*^*Cre/+*^;*Spop*^*f/f*^ mice with *Sufu*^*f/f*^ mice to generate embryos harboring all allelic combinations. To confirm the efficient targeting of *Sufu* and *Spop*, we fluorescently isolated GFP^−^expressing mesenchymal cells from mutant (*Bapx1*^*Cre/+*^;*ROSA26*^*mT/mG*^;*Sufu*^*f/f*^;*Spop*^*f/f*^) and control (*Bapx1*^*Cre/+*^;*ROSA26*^*mT/mG*^) embryos. qPCR using primers specific to the floxed deletion sites of our *Sufu* and *Spop* alleles demonstrated a relative fold expression change of 0.0065 ± 0.25 (Mean ± SEM, *n* = 3 samples, *p* = 2.10E-4; two-tailed, unpaired *t*-test) and 0.016 ± 0.11 (*n* = 3 samples, *p* = 2.00E-5; two-tailed, unpaired *t*-test), respectively, in mutant vs. control mesenchyme (Supplementary Fig. 2E). We additionally performed smFISH in *Bapx1*^*Cre/+*^;*ROSA26*^*mT/mG*^;*Sufu*^*f/f*^;*Spop*^*f/f*^ embryos shortly after Cre expression and found significant loss of *Sufu* and *Spop* in the GFP^+^ mutant mesenchyme, while expression remained intact in the adjacent epithelium (Supplementary Fig. 2B, D).

Homozygous loss of *Spop* in *Sufu* mesKO background (*Sufu Spop* mesKO) exacerbated the mesenchymal hyperplasia and epithelial reduction seen in *Sufu* mesKO mutants, while heterozygous loss of *Spop* (*Sufu* mesKO;*Spop* Het) did not significantly affect either (Fig. 2c, d, h–j). The gut weight of *Sufu* mesKO;*Spop* Hets remained comparable to controls, while *Sufu Spop* mesKOs weighed significantly more, likely due to increased mesenchymal hyperplasia (Supplementary Fig. 3B). Thus decreased pancreatic volume, normalized to gut weight, in *Sufu Spop* mesKOs may be due to decreased epithelium as well as increased mesenchyme. Further, flow cytometry analyses demonstrated a significant increase in mutant mesenchymal makeup (Supplementary Fig. 3C, D). Despite exacerbation of these defects, lineage specification remained intact in all mutant genotypes (Supplementary Fig. 3E-H).

qPCR analysis of *Sufu* mesKO;*Spop* Het and *Sufu Spop* mesKO pancreatic mesenchyme demonstrated increasing expression of Hh target, *Ptch1*, with increasing loss of *Spop* (Fig. 2e). While *Sufu* mesKO;*Spop* Het mutants exhibited similar INS^+^:GCG^+^ ratios to *Sufu* mesKOs, *Sufu Spop* mesKOs exacerbated the reduction in this ratio (Fig. 2k–o). Further measurements of somatostatin (SST)^+^ and ghrelin (GHR)^+^ cell proportions did not show significant changes (Supplementary Fig. 4F-K). It is notable that while *Sufu Spop* mesKO mutants have significantly decreased pancreatic volume, the proportion of Chromogranin A^+^ endocrine cells to pancreatic area was not significantly changed (Supplementary Fig. 4C–E). To assess the exocrine/ductal compartment, we quantified the proportion of pancreatic area made up of AMY^+^ acinar cells or MUC1^+^ ductal cells and found that AMY^+^ area in *Sufu Spop* mesKOs was significantly reduced, while MUC1^+^ proportions were not changed (Supplementary Fig. 4L–O). This relative reduction in acinar area likely reflects the abnormal expansion of mesenchymal cells surrounding mutant acini (Supplementary Fig. 3C, D).

These results demonstrate that *Sufu* and *Spop* genetically interact to offer multiple layers of Hh regulation where *Sufu* is sufficient in the absence of *Spop* but not vice versa. Further, these results show that organ size and INS^+^:GCG^+^ makeup are sensitive to the increased levels of signaling caused by loss of *Spop* in *Sufu* mesKO background.

### Mesenchymal *Sufu* and *Spop* regulate early progenitor growth

**Fig. 3:**
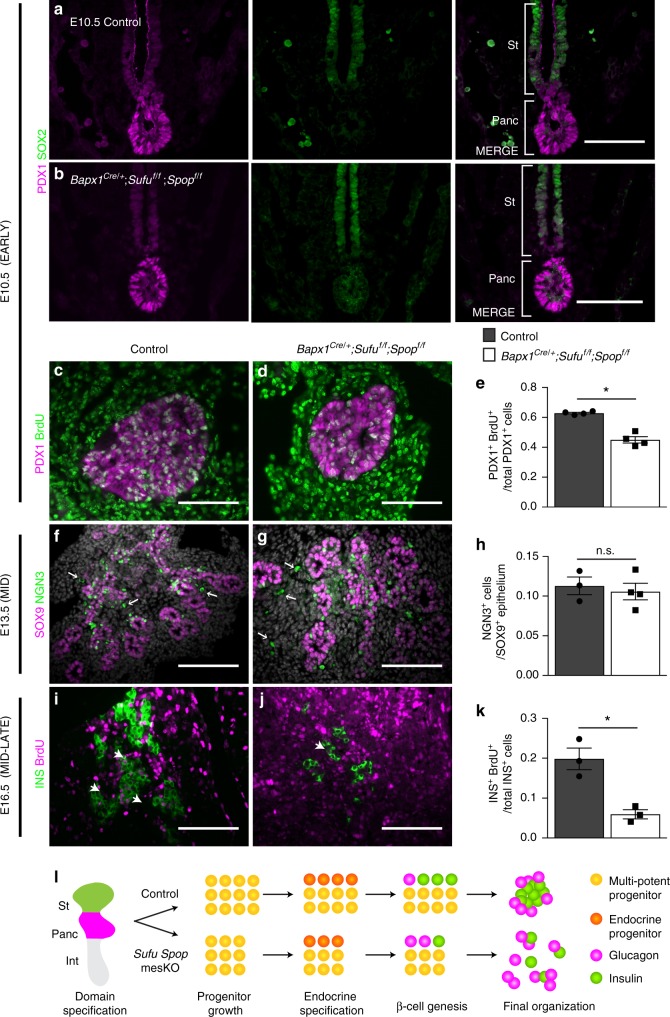
Mesenchymal loss of *Sufu* and *Spop* impairs progenitor growth and beta cell genesis. **a**, **b** Immunostaining for the SOX2^+^ stomach domain (St) and PDX1^+^ pancreatic domain (Panc) in E10.5 control **a** and *Bapx1*^*Cre/+*^;*Sufu*^*f/f*^;*Spop*^*f/f*^ mutants **b** embryos. **c**, **d** Immunostaining for E10.5 PDX1^+^ control **c** vs. mutant **d** pancreatic progenitors marked with thymidine analog, 5-bromo-2′-deoxyuridine (BrdU) 1 h after administration. **e** Assessment of the proportion of PDX1^+^ progenitors marked by BrdU as a measure of progenitor proliferation in mutants vs. controls (*n* = 4 samples per genotype). **f**, **g** Immunostaining for E13.5 NGN3^+^ control **f** vs. mutant **g** endocrine progenitors in the SOX9^+^ pancreatic progenitor pool. Arrows indicate non-specific staining. **h** Quantification of the proportion of NGN3^+^ committed endocrine progenitors out of the total NGN3^+^ and/or SOX9^+^ progenitor pool in mutants vs. controls (*n* = 3 control samples, *n* = 4 mutant samples). **i**, **j** Immunostaining for control **i** vs. mutant **j** INS^+^ cells labelled with BrdU after administration every 24 h during beta cell genesis (E14.5-E16.5). Arrows indicate representative BrdU^+^ labelled INS^+^ cells. **k** Assessment of INS^+^ cells labelled with BrdU after E14.5-E16.5 tracing as a measure of beta cell genesis in mutants vs. controls (*n* = 3 samples per genotype). **l** Diagram illustrating the temporal roles of mesenchymal *Sufu* and *Spop* throughout pancreatic development. Data are means ± SEM. n.s. denotes not significant, * denotes *p* < 0.05 by Student’s un-paired, two tailed *t*-test. Scale bars- 100 µm

It has previously been shown that final pancreas size is already determined by the size of the originating PDX1^+^ progenitor pool^34^. The pancreatic size reduction observed in *Sufu Spop* mesKOs indicates that mesenchymal Hh signaling may contribute as a determinant of final organ size. To determine if this defect originates from improper pancreatic specification, we analyzed *Sufu Spop* mesKOs during domain specification (E9.5-E10.5). Immunostaining for pancreatic TF, PDX1, and stomach TF, SOX2, demonstrated comparable domain formation of both organs, indicating that the pancreas can be properly specified in the presence of activated mesenchymal Hh signaling (Fig. 3a, b, Supplementary Fig. 6A-C). Domain specification is followed by rapid expansion of multi-potent progenitors^34^. To assess progenitor growth, we quantified the proportion of PDX1^+^ progenitors marked with proliferation marker, 5-bromo-2′-deoxyuridine (BrdU), and found significantly impaired proliferation in *Sufu Spop* mesKO pancreata (Fig. 3c–e). These results demonstrate that mesenchymal Hh regulation influences the growth of PDX1^+^ multi-potent progenitors to regulate final organ size.

### Mesenchymal *Sufu* and *Spop* are critical for beta cell genesis

Mid-pancreatic development is characterized by lineage specification (by E13.5), followed by rapid differentiation for the remainder of embryogenesis. To assess if defects in endocrine specification contribute to beta cell impairments in *Sufu Spop* mesKOs, mutant pancreata were analyzed during mid-gestation. E13.5 *Sufu Spop* mesKOs already exhibit mesenchymal hyperplasia and loss of an external pancreas (Supplementary Fig. 6D, E). Immunostaining revealed that while PDX1^+^ SOX9^+^ pancreatic progenitors are reduced (Supplementary Fig. 6H, I), the proportion of cells committed to the NGN3^+^ endocrine lineage from total SOX9^+^ progenitors remains consistent between controls and mutants (Fig. 3f–h).

Expanding on this, the segregation of an endocrine-ductal progenitor trunk vs. acinar progenitor tip domain has been demonstrated to be critical for pancreatic cell-fate allocation^35,36^. This segregation is reflected in the restriction of early progenitor marker, NKX6.1, to the bipotent trunk by E13.5^37,38^. To determine if *Sufu Spop* mesKO endocrine impairments were due to improper trunk segregation, we assessed the ratio of NKX6.1^+^ cells to SOX9^+^ progenitors and found comparable proportions and proper localization of NKX6.1^+^ cells to the SOX9^+^ population core (Supplementary Fig. 6J-L). To determine if the impaired growth of these NKX6.1^+^ trunk progenitors or of NGN3^+^ endocrine progenitors affects endocrine development, we assessed their proportions of BrdU^+^ proliferating cells and found no significant difference (Supplementary Fig. 6M-O, P-R). Together, these results indicate that endocrine commitment can occur properly in the presence of activated mesenchymal Hh signaling.

As beta cell defects in *Sufu Spop* mesKOs were not due to impaired lineage specification, we hypothesized that mesenchymal *Sufu* and *Spop* are required for the subsequent differentiation of beta cells. Sander *et al*. previously demonstrated that the intrinsic replication rate of pre-existing beta cells during mid-development is extremely low^39^. Thus, to assess beta cell formation in *Sufu Spop* mesKOs, pregnant dams were injected with BrdU every 24 h during beta cell genesis (E14.5-E16.5). With this scheme (Supplementary Fig. 7A), labelling of INS^+^ cells with BrdU should predominantly trace progenitors that have differentiated into beta cells. The proportion of INS^+^ cells co-labelled with BrdU was significantly lower in *Sufu Spop* mesKOs, demonstrating impaired beta cell genesis in the presence of activated mesenchymal Hh signaling (Fig. 3i–k). These defects appeared to be specific to beta cells, as the proportion of labelled AMY^+^ acinar, SOX9^+^ ductal, and GCG^+^ glucagon cells remained comparable (Supplementary Fig. 7B-D).

E12.5-E13.5 marks a critical turning point not only in the pancreatic epithelium but also in the gastrointestinal mesenchyme with mesenchymal cell differentiation^9,10^. Given our findings that *Sufu Spop* mesKO pancreatic mesenchyme gains gastrointestinal markers at E17.5 (Supplementary Fig. 3I-N), we hypothesized that it is at this turning point that *Sufu* and *Spop* are crucial for regulating mesenchymal identity. In E12.5 controls, SMA and SM22 are found in the stomach mesenchyme and blood vessels in the pancreas (Supplementary Fig. 6F). In contrast, *Sufu Spop* mesKO pancreatic mesenchyme stains strongly with these gastrointestinal mesenchymal markers (Supplementary Fig. 6G). These results demonstrate that without Hh regulation, pancreatic mesenchyme follows a gastrointestinal-like fate, leading to impaired progenitor growth and beta cell genesis (Summary in Fig. 3l).

### Epithelial *Sufu* and *Spop* are dispensable in pancreatogenesis

Despite this inhibitory role of mesenchymal Hh signaling in pancreatic development, the requirement for active signaling in pancreatic epithelium was previously demonstrated in *Pdx1*^*Cre/+*^;*Smo*^*f/f*^ mice^16^. Therefore, we investigated the potential role of Hh regulation by *Sufu* and/or *Spop* in pancreatic epithelium by generating *Pdx1*^*Cre/+*^;*Sufu*^*f/f*^;*Spop*^*f/f*^ mice. At E17.5, *Pdx1*^*Cre/+*^;*Sufu*^*f/f*^;*Spop*^*f/f*^ embryos exhibited no discernible phenotype, with exocrine and endocrine markers properly expressed in an organized manner (Supplementary Fig. 8A-F). Mutant mice survived into adulthood. These results demonstrate that regulation of Hh signaling by *Sufu* and *Spop* occurs in a tissue-specific manner and is not critical in the embryonic pancreatic epithelium.

### Loss of mesenchymal Hh signaling leads to ectopic branching

The analysis of *Ptch*^*LacZ*^ mice implies active signaling in both the pancreatic epithelium and mesenchyme^16^. Since epithelial-specific inhibition of Hh signaling led to milder defects than those found with global inhibition^16,17^, we hypothesized that active signaling is also required in the pancreatic mesenchyme. To address this, we generated *Bapx1*^*Cre/+*^;*Smo*^*f/f*^ mice to inhibit mesenchymal Hh signaling (Supplementary Fig. 9A, B). *Bapx1*^*Cre/+*^;*Smo*^*f/f*^ mutants demonstrated proper epithelial differentiation, as shown by the proper localization of INS and AMY at E17.5 (Supplementary Fig. 9D). Strikingly, *Bapx1*^*Cre/+*^;*Smo*^*f/f*^ embryos exhibited ectopic pancreatic branching with 100% penetrance (Supplementary Fig. 9B, C, *n* = 20 embryos). These ectopic branches extended from the ventral pancreas to form an annulus around the intestine, a phenotype resembling human congenital disorder, annular pancreas^40^. Whole-mount immunofluorescence revealed that these ectopic PDX1^+^ branches are already present at E13.5, indicating dysregulation of branching morphogenesis (Supplementary Fig. 9E, F). Thus, these results suggest that the maintenance of low-level mesenchymal Hh signaling is critical for pancreatic morphogenesis.

### *Sufu* and *Spop* mutant defects occur through mesenchymal *Gli2*

**Fig. 4:**
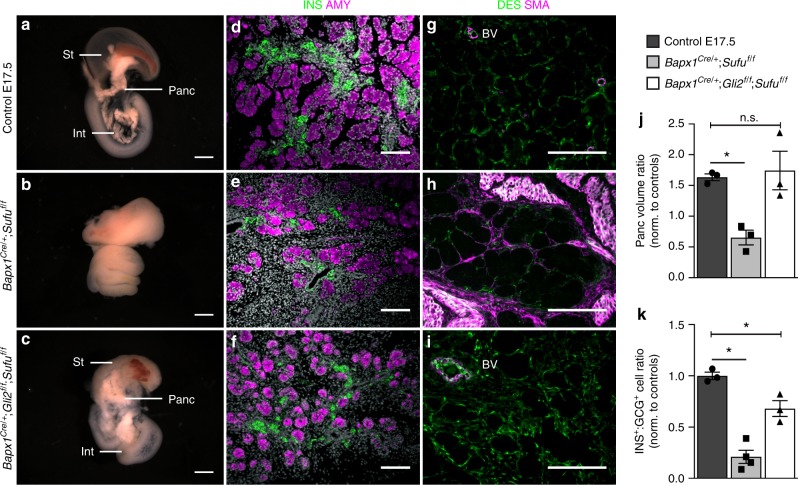
Mesenchymal deletion of *Gli2* ameliorates pancreatic defects in *Sufu* mesKOs. **a**–**c** Representative E17.5 control **a**, *Bapx1*^*Cre/+*^;*Sufu*^*f/f*^ mutant **b**, and *Bapx1*^*Cre/+*^;*Gli2*^*f/f*^;*Sufu*^*f/f*^ rescue gut **c**. St- Stomach, Int- Intestine, Panc- Pancreas. **d**–**f** Immunostaining for INS and AMY in control E17.5 **d**, *Bapx1*^*Cre/+*^;*Sufu*^*f/f*^ mutant **e**, and *Bapx1*^*Cre/+*^;*Gli2*^*f/f*^;*Sufu*^*f/f*^ rescue pancreata **f**. **g**–**i** Co-staining for DES and SMA in E17.5 control **g**, *Bapx1*^*Cre/+*^;*Sufu*^*f/f*^ mutant **h**, and *Bapx1*^*Cre/+*^;*Gli2*^*f/f*^;*Sufu*^*f/f*^ pancreatic mesenchyme **i**. BV- Blood vessel. **j** Comparison of the ratios in E17.5 pancreatic volume between *Bapx1*^*Cre/+*^;*Sufu*^*f/f*^ mutants and their controls vs. *Bapx1*^*Cre/+*^;*Gli2*^*f/f*^;*Sufu*^*f/f*^ rescues and their controls (*n* = 3 samples of each genotype). **k** Comparison of INS^+^ to GCG^+^ cell ratios in *Bapx1*^*Cre/+*^;*Sufu*^*f/f*^ and *Bapx1*^*Cre/+*^;*Gli2*^*f/f*^;*Sufu*^*f/f*^ pancreata, normalized to controls (*n* = 3 control and rescue samples, *n* = 4 mutant samples). Data are means ± SEM. n.s. denotes not significant, * denotes *p* < 0.05 by Student’s un-paired, two tailed *t*-test. Scale bars: Whole mount- 1 mm, Immunofluorescence- 100 µm

To further understand the downstream mechanisms of mesenchymal Hh regulation, we focused on GLI TFs, the pathway’s transcriptional effectors. Our qPCR analysis demonstrated the upregulation of *Gli1-3* in *Sufu* mesKO mesenchyme (Fig. 2e). GLI2 was of particular interest, as it is known as the pathway’s primary activator and is the main effector of Hh signaling in the intestine^41^. Further, GLI3 is reported to primarily be a transcriptional repressor, while *Gli1* is not required for embryogenesis but serves to potentiate active signaling^42^. Thus, we hypothesized that lowering levels of *Gli2* should ameliorate Hh-related defects most effectively. To address this, we crossed *Sufu*^*f/f*^ and *Bapx1*^*Cre/+*^;*Gli2*^*f/f*^ mice to generate *Bapx1*^*Cre/+*^;*Gli2*^*f/f*^;*Sufu*^*f/f*^ embryos (*Gli2 Sufu* mesKO). Knockout of *Gli2* in *Sufu* mesKO background reduced mesenchymal hyperplasia and led to the striking re-appearance of an external pancreas by E17.5 (Fig. 4a–c). The ratio of pancreatic volume, normalized to controls, was significantly recovered as compared to the loss seen in *Sufu* mesKO embryos (Fig. 4j). Further, the balance of INS^+^ to GCG^+^ cells was recovered to levels comparable to controls (Fig. 4d–f, k). Addressing the final defect of *Sufu* mesKOs, the pancreatic mesenchyme of *Gli2 Sufu* mesKOs returned to being DES^+^ SMA^−^ as in controls, with the restriction of SMA to blood vessels (Fig. 4g–i). The amelioration of all *Sufu* mesKO defects with loss of *Gli2* demonstrates that *Sufu* mesKO defects occur through *Gli2*-mediated Hh signaling.

To investigate if the exacerbated defects of *Sufu Spop* mesKOs could be similarly rescued, we generated *Bapx1*^*Cre/+*^;*Gli2*^*f/f*^;*Sufu*^*f/f*^;*Spop*^*f/f*^ embryos (*Gli2 Sufu Spop* mesKO). Interestingly, *Gli2 Sufu Spop* mesKOs did not exhibit external pancreata, and pancreas volume was not significantly recovered (Supplementary Fig. 10A-F). However, the endocrine makeup of insulin and glucagon cells was recovered back to levels comparable to controls, with beta cell replication left intact (Supplementary Fig. 10G-L). While *Gli2 Sufu Spop* mesKOs did not exhibit the same extent of rescue as *Gli2 Sufu* mesKOs, *Gli2 Sufu Spop* mesKOs morphologically resembled their less severe *Sufu* mesKO counterparts (Fig. 2b). These significant but incomplete rescues of *Sufu Spop* mesKO defects indicate that loss of *Gli2* alone is not able to fully restore normal levels of signaling, likely due to the activating actions of other GLI TFs. Overall, these rescue models demonstrate the dosage-sensitive response of the pancreatic epithelium to mesenchymal Hh signaling.

### Mesenchymal *Sufu* and *Spop* establish the embryonic gut niche

**Fig. 5:**
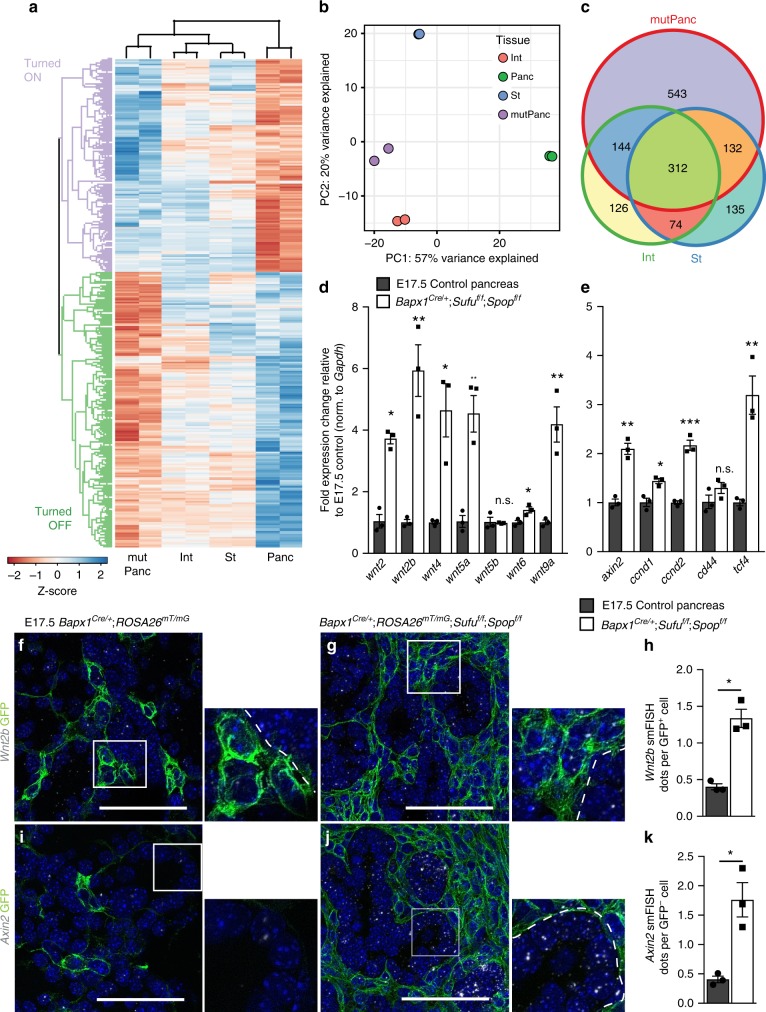
*Sufu Spop* mesKO pancreatic mesenchyme shifts towards a gastrointestinal-like signature. **a** Unsupervised hierarchical clustering analysis of all significantly differentially expressed genes in E13.5 *Bapx1*^*Cre/+*^;*ROSA26*^*mT/mG*^;*Sufu*^*f/f*^;*Spop*^*f/f*^ mutant pancreatic mesenchyme (mutPanc) and control pancreatic (Panc), stomach (St), and intestinal (Int) mesenchyme. Plot is scaled by the Z-score of log-scaled DESeq2 normalized counts, with increasing values (from red to blue) indicating relative enrichment. **b** Principal component analysis demonstrating that the mutant pancreatic mesenchyme clusters significantly closer to stomach and intestinal mesenchyme as opposed to control pancreatic mesenchyme. **c** Venn diagram representing common and unique significantly differentially regulated genes between *Bapx1*^*Cre/+*^;*ROSA26*^*mT/mG*^;*Sufu*^*f/f*^;*Spop*^*f/f*^ pancreatic mesenchyme vs. control stomach and intestinal mesenchyme. **d**, **e** qPCR analysis of Wnt ligand (**d**) and Wnt target gene (**e**) expression in E17.5 *Bapx1*^*Cre/+*^;*ROSA26*^*mT/mG*^; *Sufu*^*f/f*^;*Spop*^*f/f*^ vs. control whole pancreata (*n* = 3 samples each genotype). **f**, **g** Single molecule fluorescent in situ hybridization (smFISH) for Wnt ligand, *Wnt2b*, in E17.5 control (**f**) vs. *Bapx1*^*Cre/+*^;*ROSA26*^*mT/mG*^;*Sufu*^*f/f*^;*Spop*^*f/f*^ (**g**) pancreata co-stained with GFP to mark *Bapx1*-expressing mesenchyme. **h** Quantification of average *Wnt2b* smFISH transcripts per GFP^+^ mesenchymal cell in E17.5 mutants vs. controls (*n* = 3 samples each genotype). **i**, **j** smFISH for Wnt target gene, *Axin2*, in E17.5 control (**i**) vs. *Bapx1*^*Cre/+*^;*ROSA26*^*mT/mG*^;*Sufu*^*f/f*^;*Spop*^*f/f*^ (**j**) pancreata co-stained with GFP to mark *Bapx1*-expressing mesenchyme. **k** Quantification of average *Axin2* smFISH transcripts per GFP^-^ epithelial cell in E17.5 mutants vs. controls (*n* = 3 samples each genotype). White boxes correspond to zoomed-in inserts. Dashed outline denotes border between epithelium and mesenchyme. Data are means ± SEM. n.s. denotes not significant, * denotes *p* < 0.05, **denotes *p* < 0.005, *** denotes *p* < 0.005 by Student’s un-paired, two tailed *t*-test. Scale bars: 100 µm

To investigate the requirement for Hh signaling regulation in the pancreatic niche in an unbiased manner, we analyzed the transcriptome of sorted GFP^+^ pancreatic mesenchymal cells from E13.5 *Bapx1*^*Cre/+*^;*ROSA26*^*mT/mG*^;*Sufu*^*f/f*^;*Spop*^*f/f*^ mutants and compared this with our control digestive organ mesenchymal cells. Unsupervised hierarchical clustering analysis and PCA performed using the same 1000 most-variable genes previously identified between control mesenchymes demonstrated a clear shift of *Sufu Spop* mesKO pancreatic mesenchyme along PC1 towards the stomach and intestinal mesenchyme (Fig. 5a, b). We broadly categorized control vs. mutant pancreatic mesenchymal DE genes based on fold change values: Genes normally downregulated in the pancreatic mesenchyme but upregulated with Hh activation are denoted as Turned ON, whereas genes normally enriched but downregulated upon Hh activation are denoted as Turned OFF (Fig. 5a). Consistent with this, Hh targets were enriched within the Turned ON group (*p* < 1.55E-15; hypergeometric test with Bonferroni correction). We also identified 1131 DE genes between the control and mutant pancreatic mesenchyme, over half of which were also DE between the control pancreas vs. stomach or intestinal mesenchyme (Fig. 5c). Similarly, we observed considerable overlap between enriched GO terms between mutant DE clusters, and pancreas vs. stomach/intestine DE clusters. For example, Turned ON genes enriched for terms related to muscle development whereas Turned OFF genes enriched for terms related to neurogenesis and angiogenesis, as was observed for our Pancreas OFF and Pancreas ON clusters, respectively (Supplementary Data 4). Altogether, these analyses indicate that the transcriptional signature of mutant pancreatic mesenchyme shifts towards a more gastrointestinal-like signature.

### Mesenchymal Hh activation leads to abnormal Wnt expression

Given this shift, we hypothesized that the attainment of a more gastrointestinal-like identity in *Sufu Spop* mesKO pancreatic mesenchyme may lead to the improper activation of gastrointestinal niche factors that impair pancreatic beta cell development. Our rescue studies demonstrated that *Sufu* and *Spop* regulation occur through *Gli2* (Fig. 4, Supplementary Fig. 10). In the intestine, mesenchymal *Gli2* is critical for epithelial Wnt activity^41^. Recent studies have further found Hh-active cells that express Wnt ligands as part of the intestinal stem cell niche^43,44^. Moreover, our analysis of GLI2 genomic binding domains in the intestine has demonstrated direct activation of Wnt ligands by GLI2^45^. Altogether this suggests that in the intestinal mesenchyme, Hh signaling can activate Wnt niche signals for epithelial homeostasis.

To investigate the potential dysregulation of Wnt in *Sufu Spop* mesKOs, we performed qPCR and smFISH on whole pancreata for secreted Wnt ligands and found the upregulation of various Wnts such as *Wnt2* and *Wnt2b* (Fig. 5d, f-h). Notably, the overexpression of *Wnt2* and *Wnt2b* was rescued in both our *Gli2* rescue models, supporting *Gli2*-mediated activation of Wnt signals in *Sufu Spop* mesKOs (Supplementary Fig. 10M-T, Supplementary Fig. 11A-H).

We then hypothesized that this mesenchymal Wnt ligand expression may then activate epithelial Wnt signaling, which then contributes to the epithelial dysregulation of Hh mutants. qPCR analysis revealed the upregulation of Wnt targets such as *Axin2* (Fig. 5e), which has been reported to be a transcriptional readout of active Wnt signaling in the pancreas^24,46^. smFISH for *Axin2* demonstrates increased expression in the GFP^−^ epithelium of *Sufu Spop* mesKOs (Fig. 5i–k). These results suggest that increased Wnt expression could contribute to epithelial impairments through activation of epithelial Wnt signaling.

### Wnt signaling modulation influences organoid differentiation

**Fig. 6:**
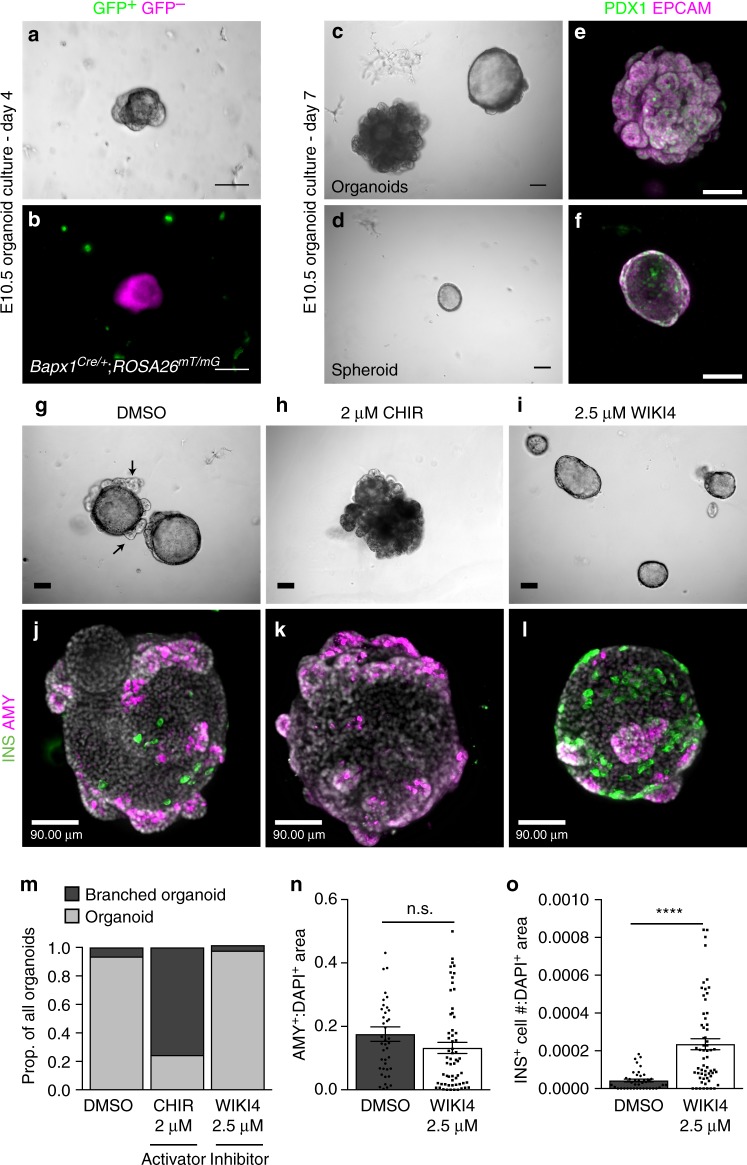
WNT signaling inhibition boosts Insulin^+^ cell development in epithelial organoids. **a**, **b** Representative brightfield (**a**) and fluorescence microscopy (**b**) images of a forming organoid established from E10.5 *Bapx1*^*Cre/+*^;*ROSA26*^*mT/mG*^ mesenchymal reporters demonstrates exclusion of GFP^+^ *Bapx1*-expressing mesenchyme. **c**, **d** Representative varieties of organoids at day 7 of culture are shown in **c**, with an extensively branched structure (left) and a more typical organoid (right). An undifferentiated spheroid is shown in **d**. **e**, **f** Organoid (**e**) and spheroid (**f**) structures formed in culture predominantly express epithelial marker, EPCAM, and pancreas marker, PDX1. **g**–**i** Morphology of organoids cultured in the presence of DMSO (**g**) vs. 2 µM WNT activator, CHIR (**h**), or 2.5 µM WNT inhibitor, WIKI4 (**i**), from day 0 to day 8. Arrows mark examples of epithelial branching projections. **j**–**l** Representative images of DMSO- (**j**), CHIR- (**k**), and WIKI4-treated (**l**) organoids stained for INS and AMY. **m** Quantification of organoids demonstrating extensive branching morphology (example in **h**), as a proportion of all organoids (*n* = 168 total organoids). **n** Immunofluorescent analysis of the ratio of AMY^+^ area to DAPI^+^ area for organoids cultured with or without 2.5 µM WIKI4 (*n* = 42 DMSO-treated organoids, *n* = 63 WIKI4-treated organoids). **o** Immunofluorescent analysis of the ratio of INS^+^ cell number to DAPI^+^ cell area (*n* = 42 DMSO-treated organoids, *n* = 63 WIKI4-treated organoids). Data are means ± SEM. n.s. denotes not significant, **** denotes *p* < 0.0005 by Student’s un-paired, two tailed *t*-test. Scale bars: Brightfield- 100 µm, Immunofluorescence- 90 µm

To define the role of Wnt signaling in the pancreatic epithelium, organoids were established from pancreatic progenitors according to work by the Grapin–Botton group^47^. Pre-organoid clusters derived from *Bapx1*^*Cre/+*^;*ROSA26*^*mT/mG*^ embryos show exclusion of GFP^+^ mesenchymal cells (Fig. 6a, b). After 7 days in culture, organoids exhibiting various levels of branching were observed along with progenitor spheroids (Fig. 6c, d). All structures observed were composed of pancreatic epithelial cells as marked by epithelial marker EPCAM, and pancreatic marker PDX1 (Fig. 6e, f).

To test if improper activation of epithelial Wnt signaling influences beta cell development, epithelial organoids were treated with 2 µM Wnt activator, CHIR from time of seeding (day 0) until harvest at day 8. In contrast to DMSO-treated controls, which express both INS and AMY, CHIR-treated organoids only developed AMY^+^ cells and lacked INS^+^ cells (Fig. 6g, j, h, k). Notably, a greater proportion of CHIR-treated organoids developed extensive branches (Fig. 6m). These results demonstrate that improper activation of Wnt signaling in the pancreatic epithelium impairs beta cell differentiation.

We next investigated if inhibition of epithelial Wnt signaling could instead promote beta cell development. Organoids were treated with 2.5 µM Wnt inhibitor, WIKI4, from the time of seeding until harvest. Representative images of WIKI4-treated organoids are shown in Fig. 6i. The identity of these structures as organoids rather than immature spheroids was confirmed by the presence of differentiated markers, INS and AMY (Fig. 6l). Organoids treated with WIKI4 exhibited a dramatic increase in the proportion of INS^+^ cells as normalized to DAPI^+^ area (Fig. 6o). On the other hand, WIKI4-treated organoids did not show a significant difference in AMY^+^ differentiation (Fig. 6n).

Immunostaining for additional endocrine markers, GCG and SST, suggests that treatment with WIKI4 leads to an overall increase in endocrine cells (Supplementary Fig. 12A-D). Treatment with WIKI4 or CHIR tended to result in the production of smaller organoids as compared to DMSO controls (Supplementary Fig. 12E). To assess endocrine cell proliferation, organoids were treated with thymidine analog, 5-Chloro-2′-deoxyuridine (EdU), for 1–2 h prior to harvest. While organoids were largely proliferative, differentiated INS^+^, GCG^+^, and SST^+^ cells were predominantly left unlabeled in both DMSO-treated and WIKI4-treated groups indicating that the endocrine cell increase observed with WIKI4 treatment may be due to the promotion of differentiation or endocrine progenitor growth (Supplementary Fig. 12F-K). Overall, these results imply that abnormal activation of Wnt signaling induced by *Sufu* and *Spop* deletion may contribute to beta cell impairment and suggests Wnt inhibition as a candidate for improving current beta cell differentiation protocols.

### Wnt inhibition improves human beta-like cell generation

**Fig. 7:**
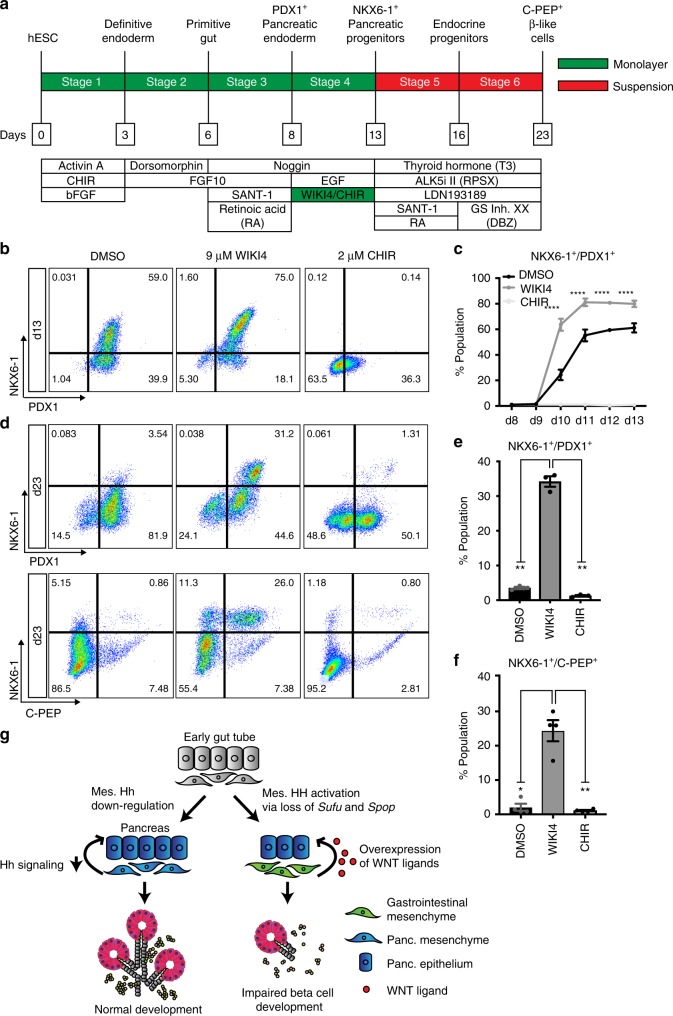
Inhibition of WNT signaling improves human stem cell to beta-like cell differentiation. **a** Schematic of hESC differentiation to beta-like cell protocol. **b** Representative flow cytometry plot of day 13 cultures differentiated with DMSO, 9 µM WIKI4, or 2 µm CHIR at stage 4. Cells were stained with anti-PDX1 and anti-NKX6-1. **c** Kinetics of NKX6-1^+^/PDX1^+^ pancreatic progenitor development from day 8 to day 13 of differentiation in the presence of DMSO, WIKI4, or CHIR (*n* = 3 independent experiments). Data are means ± SEM. **** denotes *p* < 0.0001 by two-way ANOVA with Tukey’s multiple comparison test. **d** Representative flow cytometry plot of day 23 cultures differentiated at stage 4 with DMSO, WIKI4 or CHIR. Cells were stained with anti-PDX1, anti-NKX6-1 (upper panel) and anti-C-peptide (lower panel). **e**, **f** Quantification of NKX6-1^+^/PDX1^+^ (**e**) and NKX6-1^+^/C-Peptide^+^ (**f**) percentages at day 23 of differentiation, in cultures treated with DMSO, WIKI4 or CHIR at stage 4. Data are means ± SEM. * denotes *p* < 0.05, ** denotes *p* < 0.01 by one-way ANOVA with Tukey’s multiple comparison test. **g** Down-regulation of mesenchymal Hedgehog (Hh) signaling by *Sufu* and *Spop* allows for the establishment of a pancreas-specific mesenchymal niche that properly supports epithelial development (left). Improper activation of Hh signaling due to loss of mesenchymal *Sufu* and *Spop* leads to a more gastrointestinal-like niche that secretes gastrointestinal factors such as WNT ligands (right). These WNT ligands can then act on the developing pancreatic epithelium to over-activate WNT signaling and impair beta cell differentiation

To evaluate whether human beta cell development requires Wnt inhibition, we treated hESC-derived pancreatic endoderm with WIKI4 or CHIR to inhibit or activate Wnt signaling, respectively (Schematic in Fig. 7a). Notably, kinetic analysis for PDX1 and NKX6-1 by flow cytometry from day (d)8–13 demonstrated a significant increase in the efficiency of NKX6-1^+^/PDX1^+^ pancreatic progenitor formation when cells were treated with 9 µM WIKI4, as compared to DMSO control (Fig. 7b, c, Supplementary Fig. 13A). Treatment with 2 µM CHIR almost completely inhibited NKX6-1^+^/PDX1^+^ pancreatic progenitor formation (Fig. 7b, c, Supplementary Fig. 13A). Analysis at d23 (final stage of differentiation) demonstrated an increase in the percentage of NKX6-1^+^/PDX1^+^ and NKX6-1^+^/C-PEP^+^ beta-like cells in WIKI4-treated cultures, which was confirmed by transcript analysis (Fig. 7d–f, Supplementary Fig. 13B). Importantly, the majority of these cells were C-PEP^+^/GCG^−^, with a minor percentage of poly-hormonal C-PEP^+^/GCG^+^ cells (Supplementary Fig. 14A). Surprisingly, while end stage cultures were able to release insulin granules following KCl stimulation, they failed to respond to glucose challenge, suggesting that they may require further stimuli to achieve full maturation (Supplementary Fig. 14B). We further monitored the expression of additional endocrine markers and confirmed that maturation markers such as MAFA and UCN3 are expressed at low levels as compared to human islets (Supplementary Fig. 14C). To demonstrate that the WIKI4 effect was not cell-line specific, we performed the same experiments using a human induced pluripotent stem cell line (BJ-iPSC-1) and demonstrated that WIKI4-treated cells give rise to higher percentages of NKX6-1^+^/PDX1^+^ pancreatic progenitors and NKX6-1^+^/C-PEP^+^ beta-like cells independently of the cell line used (Supplementary Fig. 15A-C). Altogether, our results demonstrate that inhibition of epithelial Wnt signaling significantly improves human pancreatic progenitor formation and beta-like cell production.

## Discussion

As differentiation of beta cells is influenced by dynamic niche signals in vivo, elucidation of these signaling activities is crucial for the recapitulation of beta cell development in vitro. Here we have demonstrated the establishment of the pancreatic mesenchymal niche through Wnt inhibition, mediated by Hh regulators, *Sufu* and *Spop*. Indeed, Wnt signaling inhibition was sufficient to promote beta cell generation in organoid and human stem cell cultures.

While our study has shown the significance of repressing gastrointestinal-enriched signals such as Hh and Wnt signaling, our RNA-seq analysis also identified an enrichment for terms related to neurogenesis in the pancreatic mesenchyme. Of note are members of the semaphorin, ephrin, glial-derived neurotrophic factor, and neurotrophic-tyrosine kinase families, which are classically regarded as neuronal factors but have also been individually implicated in insulin cell development or dysfunction^48–51^. As *Bapx1*^*Cre*^ labeling excludes neuronal cells in the pancreas^6^, it may be useful to explore their direct roles in hESC to beta cell differentiation.

Informed by our genomic findings, we revealed the tissue-specific roles of Hh regulators, *Sufu* and *Spop*. We demonstrate that loss of mesenchymal *Spop* is not sufficient to dysregulate pancreatogenesis but in conjunction with *Sufu* loss, exacerbates mutant defects. Interestingly, global *Spop* knockout in mouse embryos leads to increased beta cell area through the regulation of PDX1 stability^22^. As our mesenchymal *Spop* knockout produced no significant defects, this suggests that the phenotype seen with global knockout stems from the cell-autonomous actions of epithelial *Spop*. Pancreatic PDX1 has been demonstrated to directly regulate endocrine TFs and pancreatic cell-type allocation^52^. Thus, accumulation of PDX1 upon loss of epithelial *Spop* may then cause increased activation of the beta cell program, leading to increased endocrine area. In our *Sufu Spop* mesKO model, we see that *Spop* governs epithelial development through cell non-autonomous signaling, whereby *Spop* influences mesenchymal signals that then exert their effects on the epithelium. These results suggest that depending on the tissue context, *Spop* can positively or negatively regulate the beta cell program. This context-dependent functioning of *Spop* is reminiscent of recent studies demonstrating that *Spop* can promote or inhibit Hh signaling in different skeletal cell populations^20,21^.

We have further demonstrated the genetic interaction between *Sufu* and *Spop* in the pancreatic mesenchyme. In our study, *Sufu* mesKOs exhibited Hh-mediated defects whereas *Spop* mesKOs did not, indicating that *Sufu* is sufficient to regulate Hh signaling in the absence of *Spop* but not vice versa. This supports previous findings where SUFU was able to antagonize SPOP by sequestering GLI2/3 protein away from SPOP-mediated degradation^53,54^. Our rescue studies demonstrated that this interaction is mediated by *Gli2* with loss of mesenchymal *Gli2* significantly rescuing defects in *Sufu* mesKO and *Sufu Spop* mesKO mutants. Incomplete rescues observed may reflect a potentially redundant role for *Gli1*^42^ or, as recent work in the skeleton suggests, SPOP may preferentially target active GLI3 for degradation, where loss of *Spop* may allow for increased GLI3-mediated Hh activity^21^.

Previous reports have demonstrated that Wnt signaling activation in the pancreatic epithelium leads to gastrointestinal identity, implicating Wnt as a gastrointestinal factor^27^. Ectopic expression of Wnt mediators during later development leads to pancreatic hypoplasia^55^. These studies suggest that high levels of Wnt are inhibitory to pancreatogenesis, but how Wnt signaling is regulated was unknown. Our study demonstrates that upregulation of Hh signaling activates mesenchymal Wnt signals, providing mechanistic insight into interactions between the Hh and Wnt pathways.

Overall, our work has demonstrated that fine-tuning of mesenchymal Hh signaling is critical for pancreatic development, which proceeds through the inhibition of Wnt niche signals (Fig. 7g). Moreover, application of our murine studies to stem cell culture has revealed that Wnt signaling inhibition promotes human beta cell generation. A recent study has shown that endocrine clustering promotes beta cell maturation^56^. Therefore, modulation of Wnt signaling in sorted and re-aggregated insulin-expressing cells may further facilitate beta cell maturation.

## Methods

### Mouse models

All procedures involving animals were performed in compliance with the Animals for Research Act of Ontario and the Guidelines of the Canadian Council on Animal Care. The Toronto Centre for Phenogenomics (TCP) Animal Care Committee reviewed and approved all procedures conducted on animals at TCP. For staging embryos, noon of the day when vaginal plugs were first observed was designated as embryonic day (E)0.5. *Bapx1*^*Cre/+*^ mice were obtained as a gift from Dr. Warren Zimmer’s group^13^. *Bapx1*^*Cre/+*^ mice were obtained as a gift from Dr. Warren Zimmer’s group. The generation of *Pdx1*^*Cre*^*, Sufu*^*f/f*^*, Gli2*^*f/f*^*, Smo*^*tm2Amc*^, and *ROSA26*^*mT/mG*^ mice was previously described^53,57–60^. Embryonic stem cells containing a floxed *Spop* allele were generated by the trans-NIH knock-out mouse project (KOMP) and were obtained from the KOMP repository (www.komp.org). For proliferation assessment, BrdU (Roche) was injected (2 mg per 20 g body weight) into pregnant females intraperitonally 1 h prior to sacrifice. For beta cell tracing experiments, BrdU was injected (2 mg per 20 g body weight) into pregnant females intraperitonally every 24 h starting from E14.5 to E16.5. Littermates lacking Cre expression served as controls for each experiment unless otherwise specified.

### Histology and immunohistochemistry

Dissected organs were fixed in 4% paraformaldehyde overnight at 4 °C. Whole-mount images were acquired using a Leica MZ10 F stereomicroscope. Fixed tissues were either dehydrated and processed for paraffin sectioning at 5 µm or cryo-protected in 30% sucrose and snap-frozen in Optimal Cutting Temperature compound for cryo-sectioning at 8 µm. Paraffin sections were re-hydrated and stained with Harris’ Hematoxylin followed by staining with 1% alcoholic Eosin Y. Light microscopy images were acquired using a Nikon E1000 microscope and DS-Fi2 camera.

For immunofluorescence, re-hydrated paraffin sections or cryo-sections were subjected to heat-induced antigen retrieval in sodium citrate buffer. Sections were blocked with 10% goat serum in phosphate-buffered saline with 0.05% Tween-20 (PBSTw) at room temperature. Tissue sections were incubated overnight at 4 °C with the following primary antibodies prepared in blocking buffer: 1:1000 mouse anti-insulin (Sigma, I2018), 1:400 rabbit anti-insulin (Abcam, ab181547), 1:500 rabbit anti-amylase (Abcam, ab21156), 1:400 mouse anti-glucagon (Abcam, ab10988), 1:200 mouse anti-smooth muscle actin (Sigma, A2547), 1:300 rabbit anti-desmin (Abcam, ab32362), 1:300 rabbit anti-SM22 (Abcam, ab14106), 1:200 mouse anti-PDX1 (DSHB, F109-D12), 1:200 rabbit anti-SOX2 (Abcam, ab97959), 1:200 rat anti-BrdU (Abcam, 6326), 1:200 mouse anti-BrdU (BD Biosciences, 347580), 1:300 rabbit anti-SOX9 (EMD Millipore, AB5535), 1:200 mouse anti-NGN3 (DSHB, F25A1B3), 1:200 mouse anti-NKX6.1 (DSHB, F55A12), 1:300 rat anti-somatostatin (Abcam, ab30788), 1:300 rat anti-ghrelin (R&D systems, MAB8200), 1:200 mouse anti-EpCam (DSHB, G8.8), 1:200 rabbit anti-chromogranin A (ab15160). Sections were washed with PBSTw and then incubated with Alexa Fluor 488- and 568-conjugated highly cross-adsorbed secondary antibodies (Thermo Fisher Scientific) along with Hoechst nuclear staining (Cell Signaling Technology) for 1 h at room temperature. After washing, slides were mounted with Aqueous mounting media (Abcam, ab103748). Fluorescent microscopy images of sections were acquired using a Nikon E1000 microscope and DS-Qi1Mc camera.

Single-molecule fluorescent *in situ* hybridization (smFISH) was performed according to the manufacturer’s protocol for the RNAscope® Multiplex Fluorescent v2 kit (Advanced Cell Diagnostics). Probes used were as follows: Wnt2 (313601), Wnt2b (405031), Axin2 (400331). Slides were mounted with ProLong™ Gold Antifade Mountant (Invitrogen). For each experiment, RNAscope® 3-plex negative control and RNAscope® 3-plex positive control probes for *Mus muscularis* were run in parallel (Advanced Cell Diagnostics). smFISH images were acquired using the Nikon A1R-Si^+^ confocal microscope system. Z-series optical sections were collected with a step-size of 1 µm using Improvision Piezo focus drive.

### Whole mount immunofluorescence

Fixed tissues were permeabilized and blocked with 10% goat serum in phosphate buffered saline with 0.1% Triton-X (PBSTr) for 2 h at room temperature. Tissue was incubated with primary antibody prepared in blocking buffer for 3 days at 4 °C on a rocker. After primary incubation, tissues were washed in PBSTr overnight at 4 °C with rocking. Secondary antibody incubation was done for 3 days at 4 °C on a rocker. Tissues were washed in PBSTr overnight and mounted in Aqueous mounting media. Images were acquired on an Olympus IX81-DSU spinning disk confocal microscope equipped with Hamamatsu camera C9100-13 EM-CCD. Z-series optical sections were collected with a step-size of 1 µm using Mad City Labs Piezo focus drive.

### Morphometric and quantitative analyses

Acquired images were analyzed using ImageJ software (NIH). For E17.5 pancreatic volume estimation, 5 µm serial sections were collected. One in every four sequential slides were stained with hematoxylin and eosin to collect representative measurements for a quarter of whole pancreatic tissue. On each slide, the area of consistently spaced sections were measured using ImageJ, blind to genotype. Total pancreatic volume was estimated by multiplying measured area by section depth and number of consecutive sections. The ratio of mutant vs. control pancreatic volume (mm^3^) was presented normalized to gut weight (mg).

For E17.5 endocrine assessments, the same serial section approach was taken as above. In brief, 1 in every 5 sequential slides were immuno-stained for endocrine markers and the number of stained cells were counted, blind to genotype, to obtain representative measurements for a fifth of whole pancreatic tissue. Total INS^+^ to GCG^+^ cells counted for an entire sample was used to calculate the ratio of INS^+^:GCG^+^ cells. For E10.5 proliferation quantification, the proportion of PDX1^+^ BrdU^+^ co-positive cells were counted on all serial cryo-sections containing PDX1^+^ pancreatic progenitors. For E13.5 endocrine quantification, at least 3 representative sections were imaged and the amount of NGN3^+^ or NKX6.1^+^ cells was measured as a proportion of all single-positive or co-labelled SOX9^+^ pancreatic progenitor cells. For beta cell tracing experiments, the proportion of INS^+^, AMY^+^, SOX9^+^, or GCG^+^ cells co-labelled with BrdU^+^ was measured on at least 3 evenly spaced and representative sections. For smFISH experiments, the number of transcripts in at least 100 DAPI^+^ cells each from 3 representative sections was taken to calculate the average transcripts per cell for each sample.

### Flow cytometry

For mouse experiments, on the day of sorting; stomach, pancreas, and intestine of E13.5 *Bapx1*^*Cre/+*^;*ROSA26*^*mT/mG*^ embryos were micro-dissected into PBS with 2% fetal bovine serum (2% FBS) on ice. Supernatant was removed, and organs were digested in a 2:1 solution of TyrpLE^TM^ Express Enzyme (Gibco) and PBS in a 37 °C water bath. Organs were homogenized into single-cell suspensions by micropipette, and the digestion reaction was stopped with an equal volume of 2% FBS. All spins were run at 400 g in a 4 °C tabletop centrifuge. Cells were re-suspended in 2% FBS containing 1:5000 SYTOX Blue dead cell stain (Thermo Fisher Scientific) and filtered through a 45 µm cell strainer. Viable cells were sorted based on their green fluorescence (488 nm laser) using the MoFlo Astrios or MoFlo XDP (Beckman Coulter). Flow and mass cytometry was performed in the SickKids-UHN Flow and Mass Cytometry Facility. Representative gating strategy for flow sorting of GFP-expressing murine gut mesenchyme (*Bapx1*^*Cre/+*^;*ROSA26*^*mT/mG*^) is shown in Supplementary Fig. 16A.

For human embryonic stem cell (hESC) experiments, monolayer cells were dissociated into single cells using TrypLE^TM^ Express Enzyme at 37 °C. Cells were stained with Zombie Violet viability dye (BioLegend) for 20 min at room temperature and were fixed using Cytofix (BD Bioscience) for at least 30 min at room temperature, followed by resuspension in PBS with 10% FBS (FACS Buffer). For PDX1/NKX6.1 staining, samples were washed and incubated overnight at 4 °C with primary antibodies (mouse anti-NKX6.1: DSHB, F55A10, goat anti-PDX1: R&D, AF2419) in BD Perm/Wash buffer (BD Biosciences). Samples were then incubated in donkey anti-mouse AF647 and donkey anti-goat AF488 secondary antibodies for 30 min at room temperature. For NKX6.1/C-PEP staining, samples were washed and incubated overnight at 4 °C with primary antibodies (rat anti-C-PEP: DSHB, GN-ID4 and mouse anti-NKX6.1: DSHB, F55A10) in 5 mg mL^−1^ saponin overnight at 4 °C. Samples were then incubated in donkey α-mouse AF647 and goat α-rat PE secondary antibodies for 30 min at room temperature. Representative gating strategy for flow sorting of hESC experiments is shown in Supplementary Fig. 16B.

### Quantitative real time polymerase chain reaction (qPCR)

For murine embryonic cells, total RNA was isolated using a RNeasy kit (Qiagen, 74134). cDNA was synthesized with the SuperScript III First-Strand synthesis kit (ThermoFisher Scientific). The ViiA-7^TM^ real-time PCR system (Applied Biosystems) and SYBR^TM^ Green quantitative PCR mix (Applied Biosystems) were used to determine relative transcript levels. All gene expression assays were conducted in triplicate and normalized to *Gapdh*. Relative expression levels were determined using the comparative CT method (ΔΔCT). Results are expressed as mean ± SEM. *p*-values were determined using un-paired, two-tail Student’s *t*-tests assuming unequal variance. *p* < 0.05 was considered statistically significant.

For human cells, total RNA was extracted from cell pellets using PureLink RNA mini kit (Ambion, 12183025). In total 1 µg of RNA was reverse transcribed into cDNA using Superscript III reverse transcriptase (Invitrogen) and RNaseOUT ribonuclease inhibitor (Invitrogen). qPCR was performed using SsoAdvanced universal SYBR green supermix (BioRad) and the CFX Connect real-time PCR system (BioRad). Gene expression was normalized to the housekeeping gene, *TBP*, of each sample. Adult pancreas total RNA was purchased from Takara (636577, Lot#1202351A) and human adult islets (Donor age 60; BMI = 26.0; 95% purity) were used as positive controls. Human islets for research were provided by the Alberta Diabetes Institute IsletCore at the University of Alberta in Edmonton (http://www.bcell.org/human-islets) with the assistance of the Human Organ Procurement and Exchange (HOPE) program, Trillium Gift of Life Network (TGLN) and other Canadian organ procurement organizations. Islet isolation was approved by the Human Research Ethics Board at the University of Alberta (Pro00013094). All donors’ families gave informed consent for the use of pancreatic tissue in research.

Primers used are as follows (5’ to 3’): *Gapdh* (F- TCGTCCCGTAGACAAAATGG; R- GAGGTCAATGAAGGGGTCGT), *Gli1* (F- CCAAGCCAACTTTATGTCAGGG; R- AGCCCGCTTCTTTGTTAATTTGA), *Gli2* (F- CAACGCCTACTCTCCCAGAC; R- GAGCCTTGATGTACTGTACCAC), *Gli3* (F- CACAGCTCTACGGCGACTG; R- CTGCATAGTGATTGCGTTTCTTC), *Smo* (F- CCTCTCTCGGGCAAGACATC; R- AGTCTCCATCTACCTGAGCCA), *Ptch1* (F- AAAGAACTGCGGCAAGTTTTTG; R- CTTCTCCTATCTTCTGACGGGT), *Sufu* deletion (F- TGTCTTCCAGTCAGAGAACACCTT; R- GGTCCTCCGTCAGCAGCAT), *Spop* deletion (F- CCTCCACCTCCGGCAGAA; R- GGTTTACTCGCAAACACCATTTCA), *Wnt2* (F- CTCGGTGGAATCTGGCTCTG; R- CACATTGTCACACATCACCCT), *Wnt2b* (F- CGAGGTGGCAAACATCCTAT; R- CTTTGAAGGCTCCACTCCTG), *Wnt4* (F- AGACGTGCGAGAAACTCAAAG; R- GGAACTGGTATTGGCACTCCT), *Wnt5a* (F- CAACTGGCAGGACTTTCTCAA; R- CATCTCCGATGCCGGAACT), *Wnt5b* (F- GAGAGCGTGAGAAGAACTTTGC; R- GGCGACATCAGCCATCTTAT), *Wnt6* (F- GCAAGACTGGGGGTTCGAG; R- CCTGACAACCACACTGTAGGAG), *Wnt9a* (F- GGCCCAAGCACACTACAAG; R- AGAAGAGATGGCGTAGAGGAAA), *Axin2* (F- GGACTGGGGAGCCTAAAGGT; R- AAGGAGGGACTCCATCTACGC), *Cyclind1* (F- GCGTACCCTGACACCAATCTC; R- CTCCTCTTCGCACTTCTGCTC), *Cyclind2* (F-CAAGGAGGGACTCCATCTACGC; R- AGAGGAGTCCCGTGTCAGTAGG), *Cd44* (F- CACCATTGCCTCAACTGTGC; R- TTGTGGGCTCCTGAGTCTGA), *Tcf4* (F- AGCCCGTCCAGGAACTATG; R- TGGAATTGACAAAAGGTGGA), *NKX6-1* (F- AGAGGACGACGACTACAATAAGCC; R- ACTTGTGCTTCTTCAACAGCTGCG), *PDX1* (F- TACTGGATTGGCGTTGTTTGTGGC; R- AGGGAGCCTTCCAATGTGTATGGT), *INS* (F- AGAAGCGTGGCATTGTGGAACA; R- TATTCCATCTCTCTCGGTGCAGGA), *NEUROD1* (F- TCCCATGTCTTCCACGTTAAGCCT; R- CATCAAAGGAAGGGCTGGTGCAAT), *NKX2-2* (F- GACAACTGGTGGCAGATTTCGCTT; R- AGCCACAAAGAAAGGAGTTGGACC), *MAFA* (F- ATTCTGGAGAGCGAGAAGTGCCAA; R- CGCCAGCTTCTCGTATTTCTCCTT), *UCN3* (F- GAGGCACCCGGTACAGATAC; R- GAGGGACAGGGTGAACTTGG), *ISL1* (F- TCCCTATGTGTTGGTTGCGG; R- CCGCATGCCATTCCAAATCC).

### Pancreatic organoid culture

Embryonic pancreatic organoids were established based on the protocol established by Greggio et al.^47^. For the work reported herein, E10.5 dorsal pancreatic buds were micro-dissected away from the embryonic gut tube. Mesenchymal tissue surrounding the pancreatic epithelial bud was manually removed using 30-gauge needles. Epithelial buds were incubated in 0.05% trypsin-EDTA (Gibco) at 37 °C for 5 min and digestion was inactivated by transferring buds to DMEM/Nutrient Mixture F12 (DMEM/F12, Gibco) containing 10% FBS. Buds were dissociated to a homogenous cell suspension by micropipette and volume was adjusted to achieve an approximate cell density of 40 cells/µL. This cell suspension was mixed at a 1:3 ratio with growth factor-depleted Matrigel (VWR) and aliquoted at 8 µL/well in a 96-well plate. Plated Matrigel suspensions were polymerized and then cultured with media that was replaced every 3–4 days in a tissue culture incubator (37 °C, 5% CO_2_).

Base media composition was as follows: DMEM/F12, 1X Penicillin-Streptomycin (Sigma Aldrich), 10% KnockOut Serum Replacement (Gibco), 0.1 mM β-mercaptoethanol (BioRad), 16 nM phorbol-12-myristate-13-acetate (EMD Millipore), 10 µM Y-27632 dichloride (Abcam), 25 ng mL^−1^ human EGF (Sigma Aldrich); 500 ng mL^−1^ recombinant mouse R-spondin 1 (R&D Systems), 100 ng mL^−1^ recombinant mouse FGF10 (R&D Systems), and 2 U mL^−1^ heparin sodium salt (Sigma Aldrich). Final concentrations of WIKI4 (Tocris) tested were 2.5 µM and 9 µM^61^. CHIR (Tocris) was used at a final concentration of 2 µM. Corresponding Dimethyl sulfoxide (DMSO) controls were used for each experiment.

### hESC culture and differentiation

H1 hESC line was obtained from WiCell Research Institute (Madison WI, USA), BJ-iPSC1 was obtained from Drs. Toshi Araki and Ben Neel (New York University, NY, USA)^62^. These lines were obtained with informed consent. Dr. Nostro has approval from the Stem Cell Oversight Committee (Canadian Institute of Health Research) to conduct work with SCOC-approved H1 and human iPSCs. Undifferentiated hPSCs were maintained and expanded as previously described^63^. Differentiation begins at stage 0 (d0) when hPSC cultures reach 70–80% confluence. All media were supplemented with 1% glutamine (Hyclone) and 1% Penicillin-Streptomycin (Hyclone). Stage 0 (d0–d1) media consisted of RPMI (Gibco), 1% MTG, 100 ng mL^−1^ hActivin A (R&D Systems) and 2 µM CHIR990210 (Tocris) for 1 day. Stage 1 media (d1-d3) consisted of RPMI media containing 50 µg mL^−1^ ascorbic acid (Sigma), 100 ng mL^−1^ hActivin A and 5 ng mL^−1^ hbFGF (R&D system). Stage 2 media (d3-d6) consisted of RPMI media supplemented with 1% vol/vol MACS NeuroBrew-21 (without vitamin A, Miltenyi Biotec), 50 ng mL^−1^ hFGF10 (R&D System) and 0.75 µM Dorsomorphin (Sigma). Stage 3 media (d6-d8) consisted of DMEM (Gibco) supplemented with 1% vol/vol NeuroBrew, 50 µg mL^−1^ ascorbic acid, 50 ng mL^−1^ hNOGGIN (R&D Systems), 50 ng mL^−1^ hFGF10, 0.25 µM SANT-1 (Tocris) and 2 µM all-trans retinoic acid (RA) (Sigma). Stage 4 media (d8–d13) consisted of DMEM containing 1% vol/vol Neurobrew supplement, 50 µg mL^−1^ ascorbic acid, 50 ng mL^−1^ hNOGGIN and 100 ng mL^−1^ hEGF (R&D System). Cells at stage 4 were also treated with DMSO, 9 µM WIKI4 (Tocris) or 2 µM CHIR990210 (Tocris).

Cells were mechanically aggregated using a pipette on d13 and were plated in suspension at 2 million cells/mL of stage 5 media in low-adherent tissue culture plates. Stage 5 media (d13–d16) consisted of MCDB131 (Gibco) consisted of 1% vol/vol NeuroBrew supplement, 1 µM T3 (Sigma), 1.5 g L^−1^ NaHCO3_3_ (Gibco), 15 mM D-(+)-glucose (Sigma), 10 µg mL^−1^ Heparin (Sigma), 0.25 µM SANT-1, 10 µM RepSox (Tocris), 100 nM LDN193189 (Cayman), 10 µM ZnSO_4_ (Sigma), 0.05 µM all-trans RA and 10 µM Y27632 (Tocris). Stage 6 media (d16-d23/24) consisted of MCDB131 supplementing with 1 µM T3, 1.5 g L^−1^ NaHCO_3_, 1% vol/vol Neurobrew supplement, 10 µg mL^−1^ Heparin, 10 µM RepSox, 100 nM LDN193189, 10 µM ZnSO_4_, and 100 nM DBZ (Tocris). Fresh stage 6 medium was replaced on d20. Aggregates were harvested in trypsin on d23 or d24 for flow cytometry or collected as cell pellets for RNA extraction.

### Glucose stimulated insulin secretion (GSIS) assay

WIKI4-derived cells were dissociated on d20 of differentiation and allowed re-aggregation at the density of 1 million cells per mL in stage 6 media. On d23 of differentiation, these cells were further cultured until d35 or d36 in the absence of growth factors and small molecules according to Velazco-Cruz et al. ^64^. In brief, this media consisted of MCDB131 supplemented with 1% glutamine, 1% vol/vol NeuroBrew and 1.5 g/L NaHCO_3_ until GSIS assay. In total 4–5 technical replicates of ~20 aggregates originating from three different batches of cells were collected and equilibrated in KREBs buffer (pH 7.4; 0.1% FFA-free BSA) containing low glucose (2.8 mM glucose) for 1 h prior to sequential incubation in low glucose (2.8 mM glucose), high glucose (16.7 mM glucose) or KCl (16.7 mM glucose + 25 mM KCl) for 1 h each. All incubation steps were performed at 37 °C in a tissue culture incubator. Supernatant was collected and aggregates were lysed using acid-ethanol protein extraction. Insulin content in samples was measured and quantified by HTRF assay (Cisbio, 62IN2PEH).

### RNA sequencing

RNA was isolated from sorted E13.5 GFP^+^ cells using the RNeasy kit (Qiagen, 74134). The integrity of samples was determined on Agilent Bioanalyzer, RIN >8.5 was considered acceptable. Poly-A RNA libraries were generated, and cDNA libraries were sequenced in 126 bp paired-end reads on the Illumina HiSeq 2500 platform. Next-generation sequencing experiments were performed by The Centre for Applied Genomics, The Hospital for Sick Children, Toronto, Canada.

*RNA-seq data analysis*: Demultiplexed raw reads were obtained in fastq format and trimmed for quality with trimmomatic to remove adapters and low-quality reads, retaining reads with a minimum length of 36 bp. Reads with both pairs surviving were aligned to the GRCm38.68 assembly (mm10) and mouse Gencode version 4 using STAR v2.5.1b^65^. Gene level read counts were obtained using featureCounts with default settings for non-stranded libraries (Supplementary Data 1, 2)^66^.

*Differential Expression Analysis*: Normalization and differential expression analysis was performed using DESeq2^67^. PCA was done using the top 1000 most variable genes between control intestine, stomach, and pancreas datasets based on variance-stabilized counts. Hierarchical clustering was done on genes which were differentially expressed between any pair of normal tissues using z-transformed DESeq2 normalized counts and the hclust function in R with default parameters. Groups were defined by cutting the resultant tree into 4 clusters.

*Regulatory network analysis*: We constructed regulatory networks using the tool PANDAR, which infers TF-gene regulatory interactions based on co-expression and motif occurrence^68^. We collected all publicly available human and mouse TF position-weight matrices (PWMs) from cis-BP. Using these, we procured a set of mouse-centric TF gene symbols by conversion of Ensembl gene IDs from mouse, and mouse orthologues of human Ensembl gene IDs. The final set of PWMs used for scanning consisted primarily of mouse PWMs; human PWMs were used for TFs for which mouse PWMs were unavailable. We then scanned for motifs within promoter regions of DE genes, defined as 2.5 kb upstream and 500 bp downstream of any annotated TSS. Scanning was performed using matrix-scan from the RSAT suite of tools^69^. In the case of multiple promoter regions (from multiple TSS), multiple PWMs, or multiple sites, we selected the best score for use in PANDAR. Expression data consisted of 348 mouse CAGE-seq datasets profiled in Fantom5. Per-gene TPM values were obtained by summing individual values for all TSS for a given gene; universal/whole-body RNA samples were omitted. Regulatory interactions were inferred using the function panda() with default settings.

### Quantification and statistical analysis

Quantitated results are expressed as mean ± SEM. *p*-values were determined using un-paired, two-tail Student’s *t*-tests assuming unequal variance unless otherwise specified. *p* < 0.05 was considered statistically significant. Multiple measurements were taken from distinct samples to generate the reported averages. A minimum sample number of three was used for all quantification.

### Reporting summary

Further information on research design is available in the Nature Research Reporting Summary linked to this article.

## Supplementary information

Supplementary Information

Reporting Summary

Description of Additional Supplementary Files

Supplementary Data 1

Supplementary Data 2

Supplementary Data 3

Supplementary Data 4

Source Data

## Data Availability

RNA-seq data generated in this study have been deposited in GEO under the accession GSE123136. Raw fastq data is available in SRA under the accession SRP171166. Target gene lists for BMP signaling were obtained from 10.1101/gr.092114.109^70^. Source data underlying Figs. 2E, J, O, 3E, H, K, 4J, K, 5D, E, H-K, 6M-O, 7C, E, F, and Supplementary Figs, 2E, 3B, C, 4E, H, K, N, O, 6C, L, O, R, 7B-D, 10F, I, L, S, T, 11D, H, 12E, 13B, 14A-C, 15B, C are included as a Source Data File. All other relevant data supporting the key findings of this study are available within the article, in the supplementary files, or from the corresponding author upon reasonable request.
