# Supplementary material for: Sufu- and Spop-mediated downregulation of Hedgehog signaling promotes beta cell differentiation through organ-specific niche signals
Source: Nat Commun. 2019 Oct 11;10:4647. doi: 10.1038/s41467-019-12624-5 (PMC6789033; doi:10.1038/s41467-019-12624-5)
Supplement: Supplementary file 1 — Supplementary Information [file 41467_2019_12624_MOESM1_ESM.pdf]

Supplementary Information

Sufu and Spop mediated Hedgehog signaling promotes beta cell differentiation through organ specific niche signals

Yung and Poon et al.

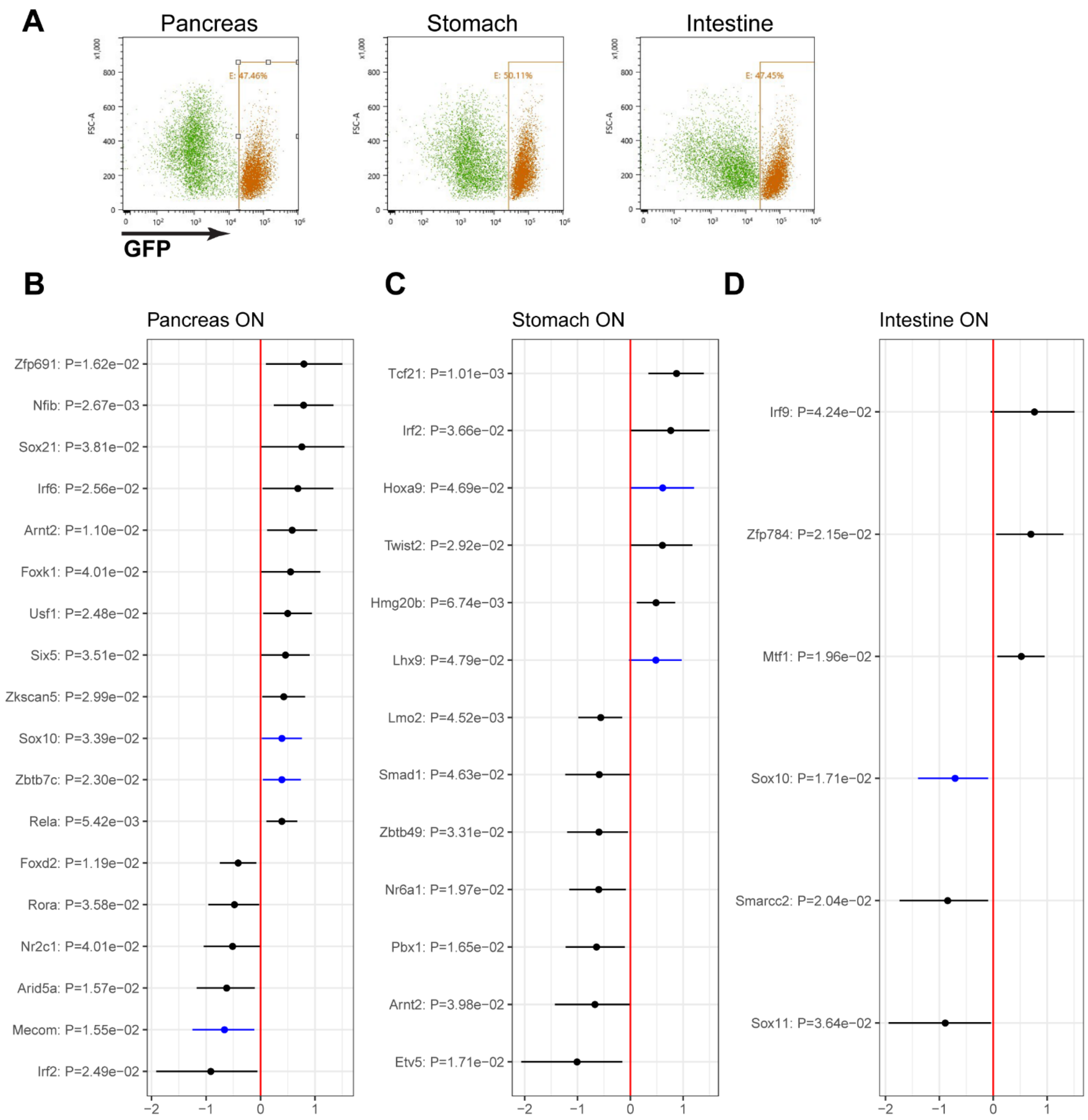

Supplementary Figure 1.

Organ-specific mesenchymal transcription factors underlie E13.5 digestive organ development. **A)** Representative flow cytometry plots of GFP^+ reporter expression in live cells of E13.5 *Bapx1*$^{Cre/+}$;*ROSA26*$^{mT/mG}$ pancreas, stomach, and intestine. Horizontal axis indicates extent of GFP fluorescence. **B-D)** Transcription factors enriched in the pancreatic (B), stomach (C), and intestinal (D) mesenchyme. Horizontal line shows 90% confidence intervals; only TFs with $p<0.05$ are shown (Fisher's exact test), TFs plotted in blue are differentially expressed between control pancreatic mesenchyme and at least one of the stomach or intestinal mesenchyme types.

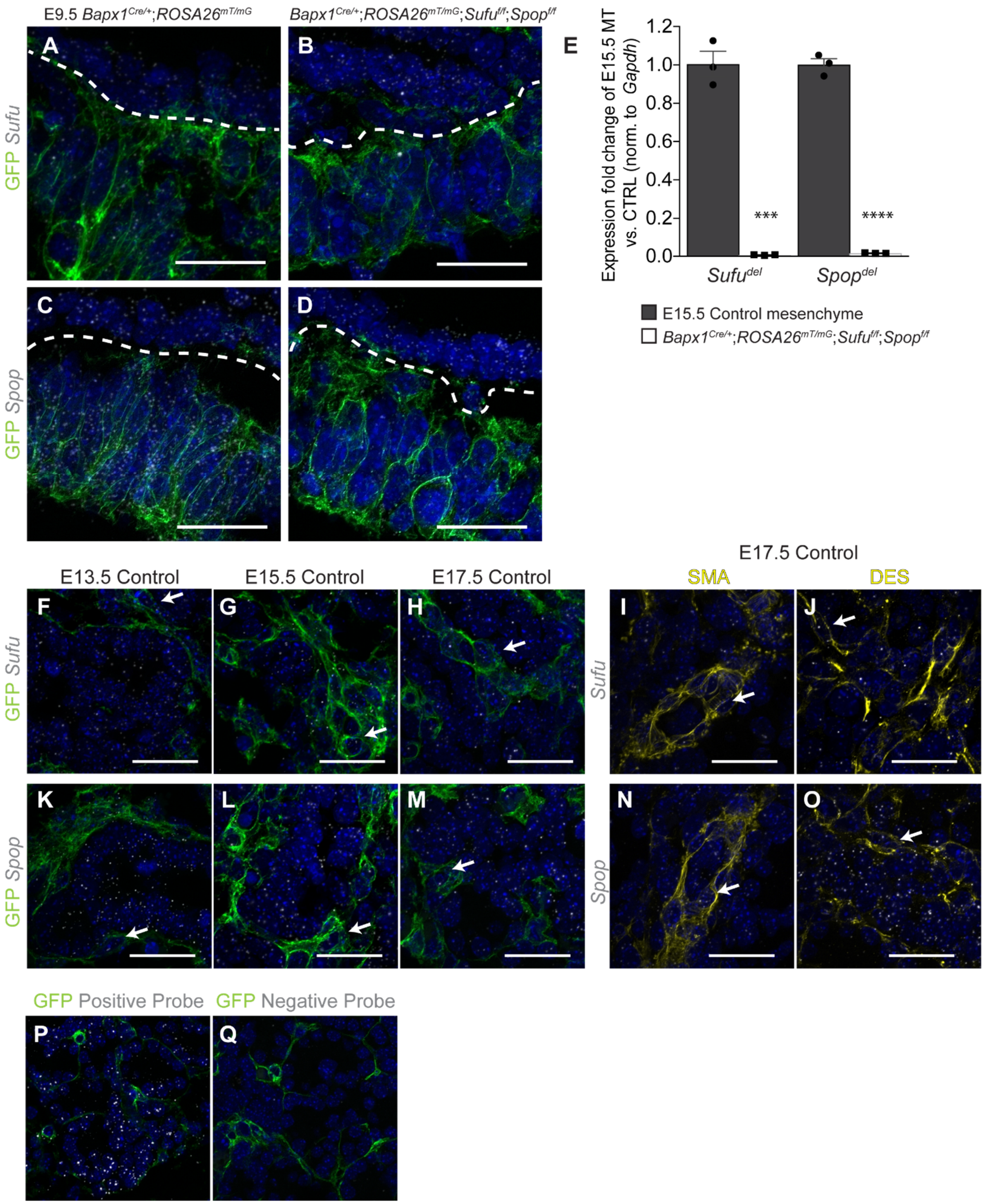

E9.5 Bapx1^Cre/+;ROSA26^mT/mG
Bapx1^Cre/+;ROSA26^mT/mG;Sufu^f/f;Spop^f/f
A
B
C
D
E
GFP Sufu
GFP Spop
Expression fold change of E15.5 MT vs. CTRL (norm. to Gapdh)
1.2
1.0
0.8
0.6
0.4
0.2
0.0
***
****
Sufu^del
Spop^del
E15.5 Control mesenchyme
Bapx1^Cre/+;ROSA26^mT/mG;Sufu^f/f;Spop^f/f
E13.5 Control
E15.5 Control
E17.5 Control
E17.5 Control
SMA
DES
F
G
H
I
J
K
L
M
N
O
Sufu
Spop
GFP Positive Probe
GFP Negative Probe
P
Q

Supplementary Figure 2.

***Sufu* and *Spop* exhibit widespread expression throughout embryonic pancreatic development.**
A-B) Single molecule fluorescent *in situ* hybridization (smFISH) for *Sufu* in $Bapx1^{Cre/+}$;$ROSA26^{mT/mG}$ control (A) versus $Bapx1^{Cre/+}$;$ROSA26^{mT/mG}$;$Sufu^{f/f}$;$Spop^{f/f}$ mutant (B) E9.5 gut tubes co-stained with GFP to mark *Bapx1*-expressing mesenchymal cells. **C-D)** smFISH for *Spop* in $Bapx1^{Cre/+}$;$ROSA26^{mT/mG}$ control (C) versus $Bapx1^{Cre/+}$;$ROSA26^{mT/mG}$;$Sufu^{f/f}$;$Spop^{f/f}$ mutant (D) E9.5 gut tubes co-stained with GFP. **E)** qPCR for the deletion sites of our *Sufu* and *Spop* alleles in sorted E15.5 GFP^+ mesenchymal cells of $Bapx1^{Cre/+}$;$ROSA26^{mT/mG}$;$Sufu^{f/f}$;$Spop^{f/f}$ vs. $Bapx1^{Cre/+}$;$ROSA26^{mT/mG}$ pancreata (n=3 samples per genotype). **F-H,K-M)** smFISH expression of *Sufu* (F-H) and *Spop* (K-M) in $Bapx1^{Cre/+}$;$ROSA26^{mT/mG}$ pancreata during endocrine specification (E13.5), beta cell differentiation (E15.5), and final organization (E17.5). Co-staining with GFP marks mesenchymal reporter cells. **I-J,N-O)** smFISH expression of *Sufu* (I-J) and *Spop* (N-O) in SMA^+ and DES^+ interstitial subpopulations. **P)** Positive control probe on E17.5 pancreas tissue co-stained with mesenchymal GFP. **Q)** Negative control probe on E17.5 pancreas tissue co-stained with mesenchymal GFP. Arrows denote representative *Sufu* or *Spop* expressing cells. Dashed lines delineate mesenchyme versus epithelium. Data are means ± SEM. n.s. denotes not significant, *** denotes p<0.005, **** denotes p<0.0005 by Student’s un-paired t-test. Scale bars- 50 μm.

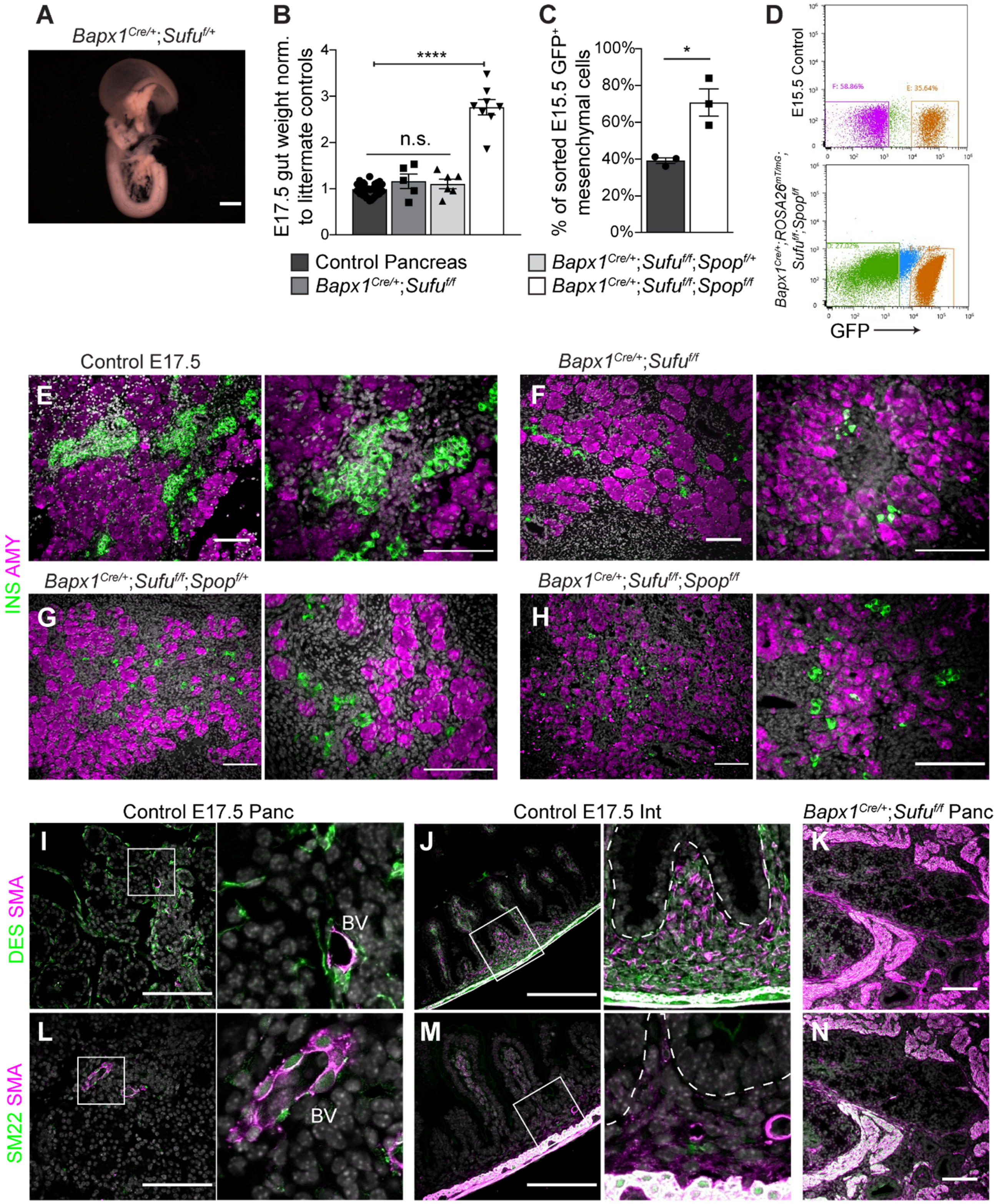
A
Bapx1Cre/+;Sufuf/+
B
E17.5 gut weight norm. to littermate controls
****
n.s.
C
% of sorted E15.5 GFP+ mesenchymal cells
100%
80%
60%
40%
20%
0%
*
D
E15.5 Control
Bapx1Cre/+;ROSA26mT/mG;Sufuf/f;Spopf/f
GFP
Control Pancreas
Bapx1Cre/+;Sufuf/f
Bapx1Cre/+;Sufuf/f;Spopf/+
Bapx1Cre/+;Sufuf/f;Spopf/f
Control E17.5
E
Bapx1Cre/+;Sufuf/f
F
INS AMY
Bapx1Cre/+;Sufuf/f;Spopf/+
G
Bapx1Cre/+;Sufuf/f;Spopf/f
H
Control E17.5 Panc
Control E17.5 Int
Bapx1Cre/+;Sufuf/f Panc
I
BV
J
K
DES SMA
L
BV
M
N
SM22 SMA

Supplementary Figure 3.

Mesenchymal loss of *Sufu* and S*pop* impairs both pancreatic epithelium and mesenchyme.
A) Representative image of E17.5 *Bapx1*$^{Cre/+}$;*Sufu*$^{f/f}$ gut. **B)** Ratio of *Sufu* and *Spop* mutant gut weight normalized to their littermate controls (n=50 controls, 5 *Bapx1*$^{Cre/+}$;*Sufu*$^{f/f}$, 6 *Bapx1*$^{Cre/+}$;*Sufu*$^{f/f}$;*Spop*$^{f/+}$, and 8 *Bapx1*$^{Cre/+}$;*Sufu*$^{f/f}$;*Spop*$^{f/f}$ samples). **C)** Percentage of GFP^{+} mesenchymal cells out of total live cells in E15.5 *Bapx1*$^{Cre/+}$;*ROSA26*$^{mT/mG}$ controls and *Bapx1*$^{Cre/+}$;*ROSA26*$^{mT/mG}$;*Sufu*$^{f/f}$;*Spop*$^{f/f}$ mutants (n=3 samples per genotype). **D)** Representative flow cytometry plots of GFP^{+} and GFP^{-} live cells in control (upper panel) and mutant (lower panel) mesenchymes used to calculate panel C. **E-H**) Immunostaining for INS and AMY in E17.5 control (E), *Bapx1*$^{Cre/+}$;*Sufu*$^{f/f}$ (F), *Bapx1*$^{Cre/+}$;*Sufu*$^{f/f}$;*Spop*$^{f/+}$ (G), and *Bapx1*$^{Cre/+}$;*Sufu*$^{f/f}$;*Spop*$^{f/f}$ (H) pancreata. Left panels: Lower magnification overview. Right panels: Higher magnification images for clarity. **I,L)** In the pancreatic mesenchyme, SMA (I) and SM22 (L) are found expressed in blood vessels (BV) with the mesenchyme being predominantly single positive for DES (I). **J,M)** Immunostaining for SMA (J) and SM22 (M) throughout the intestinal mesenchyme at E17.5. **K,N)** In *Bapx1*$^{Cre/+}$;*Sufu*$^{f/f}$ embryos, pancreatic tissue is embedded into a matrix of SMA^{+} (K) and SM22^{+} (N) cells. White boxes correspond to higher magnification panels. Data are means ± SEM. n.s. denotes not significant, * denotes $p<0.05$ by Student's un-paired t-test. Scale bars: Whole mount- 1 mm, Immunofluorescence- 100 μm.

Supplementary Figure 4

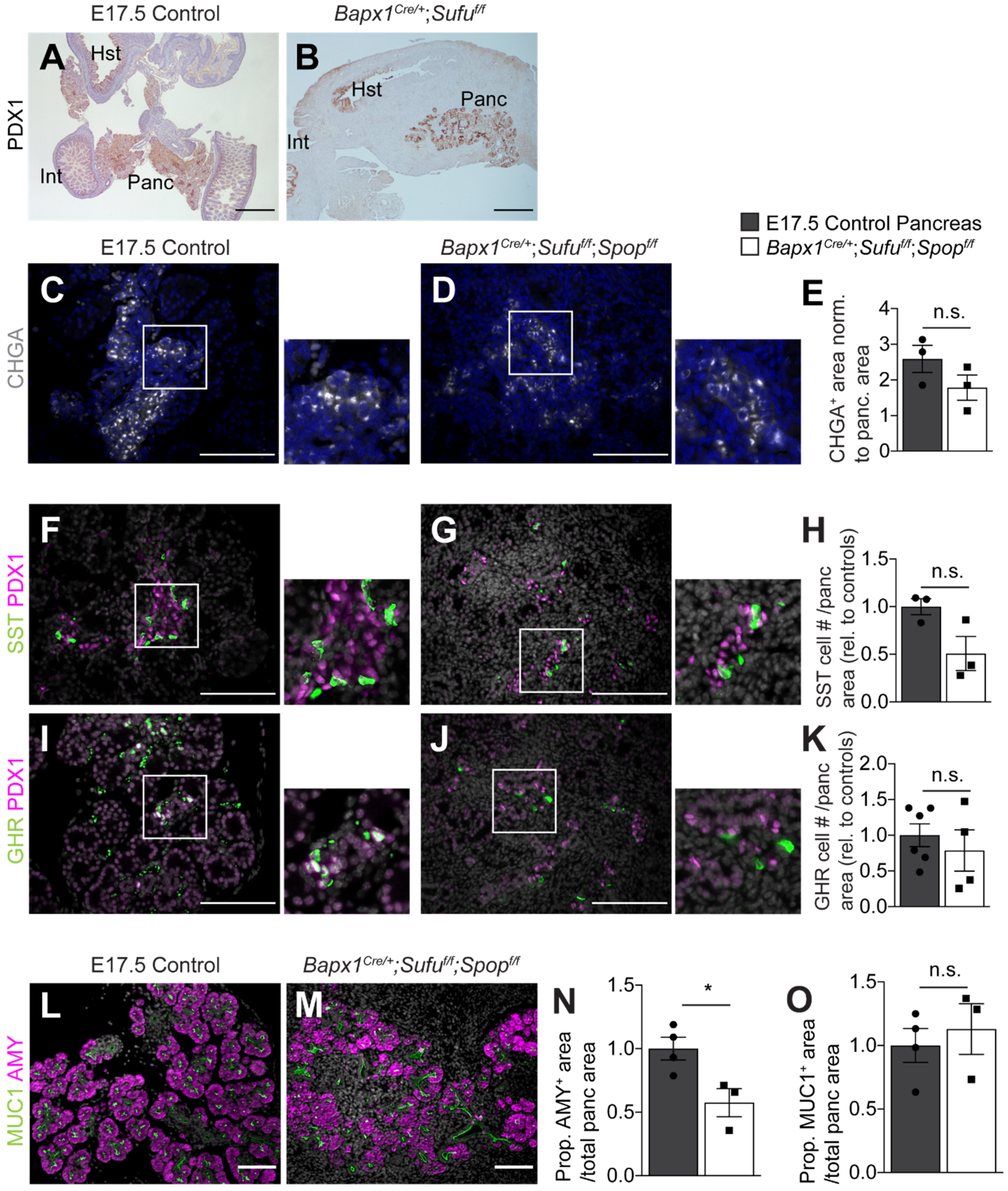

Supplementary Figure 4.

Characterization of endocrine and exocrine cell populations in *Sufu Spop* mesenchymal mutants. A-B) Immunohistochemical staining for PDX1 in the pancreas (Panc), hindstomach (Hst), and proximal intestine (Int) of E17.5 control (A) and *Bapx1*$^{Cre/+}$;*Sufu*$^{f/f}$ mutants (B). No ectopic formation of PDX1^{+} cells is observed outside of these domains. **C-D)** Immunostaining for Chromogranin A (CHGA) in E17.5 controls (C) versus *Bapx1*$^{Cre/+}$;*Sufu*$^{f/f}$;*Spop*$^{f/f}$ mutants (D). **E)** Assessment of CHGA^{+} endocrine cell area as a proportion of pancreatic tissue area (n=3 samples per genotype). **F-G)** Immunostaining for Somatostatin (SST) and PDX1 in E17.5 controls (F) versus *Bapx1*$^{Cre/+}$;*Sufu*$^{f/f}$;*Spop*$^{f/f}$ mutants (G). **H)** Quantification of SST^{+} cell numbers as normalized to pancreatic tissue area in controls versus mutants (n=3 samples each genotype). **I-J)** Immunostaining for Ghrelin (GHR) and PDX1 in E17.5 controls (I) versus *Bapx1*$^{Cre/+}$;*Sufu*$^{f/f}$;*Spop*$^{f/f}$ mutants (J). **K)** Quantification of GHR^{+} cell numbers as normalized to pancreatic tissue area in controls versus mutants (n=6 control, 4 mutant samples). **L-M)** Representative images for exocrine markers, AMY (acini) and MUC1 (ducts) in E17.5 control (L) versus *Bapx1*$^{Cre/+}$;*Sufu*$^{f/f}$;*Spop*$^{f/f}$ mutants (M). **N)** Quantification of the proportion of AMY^{+} area to total pancreatic tissue area in mutants, normalized to proportions found in controls (n=3 samples per genotype). **O)** Quantification of the proportion of MUC1^{+} area to total pancreatic tissue area in mutants, normalized to proportions found in controls (n=3 samples per genotype). White boxes correspond to higher magnification panels. Data are means ± SEM. n.s. denotes not significant, * denotes $p<0.05$ by Student's un-paired t-test. Scale bars: Histology- 500 µm, Immunofluorescence- 100 µm.

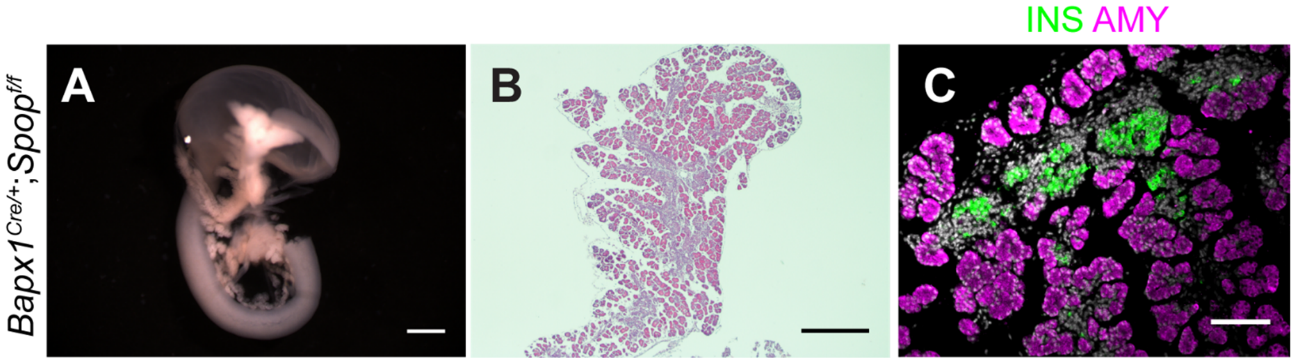

Supplementary Figure 5.

Mesenchymal loss of *Spop* does not impair pancreatic development. A) Mesenchymal loss of *Spop* (*Bapx1*$^{Cre/+}$;*Spop*$^{f/f}$) does not overtly affect pancreatic morphology. **B)** Representative histological stain of *Bapx1*$^{Cre/+}$;*Spop*$^{f/f}$ pancreas. **C)** Immunostaining for INS and AMY in E17.5 *Bapx1*$^{Cre/+}$;*Spop*$^{f/f}$ pancreas. Scale bars: Whole mount- 1 mm, Histology- 500 µm, Immunofluorescence- 100 µm.

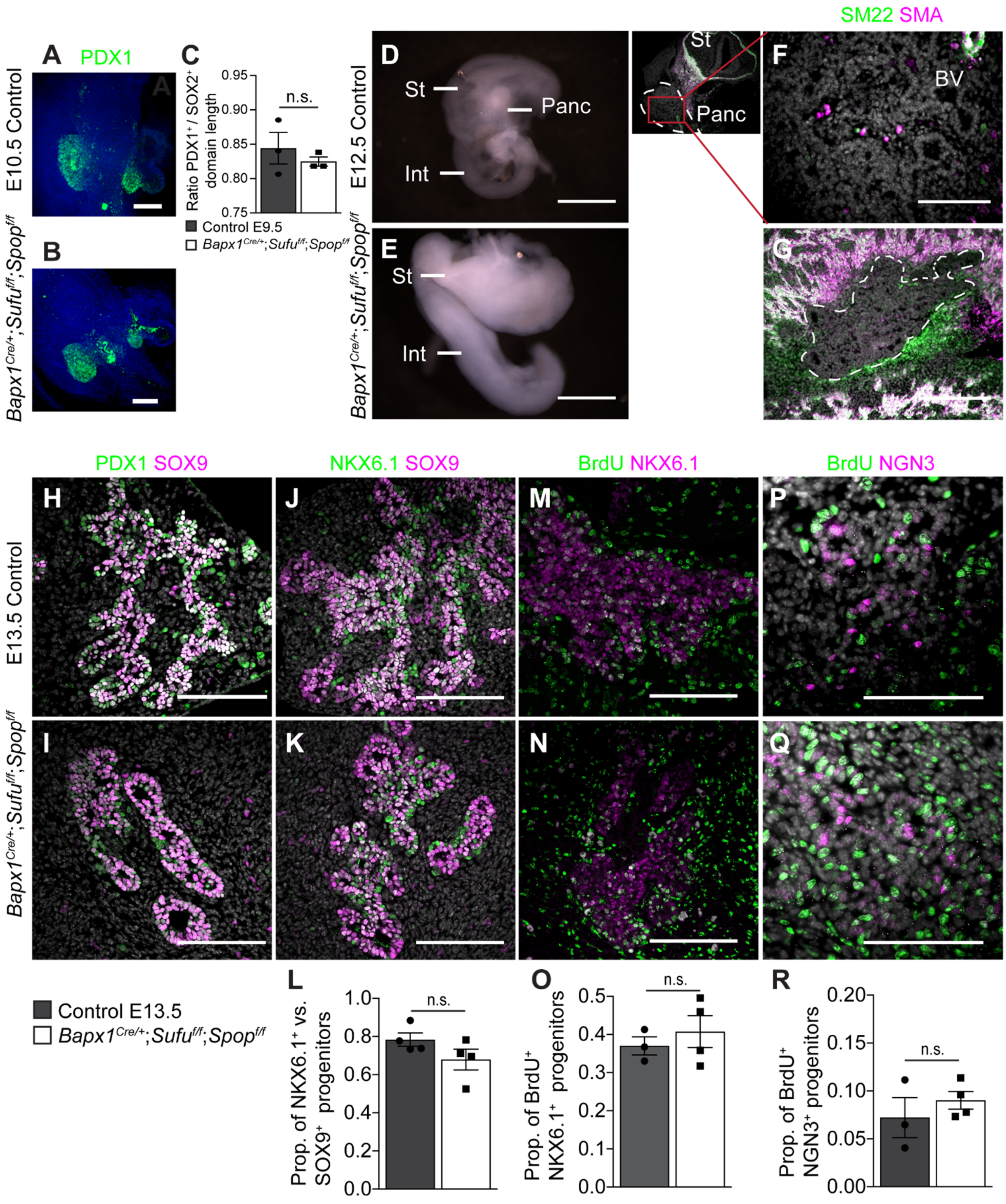

SM22 SMA
A PDX1
C
E10.5 Control
Bapx1Cre/+;Sufuf/f;Spopf/f
B
Ratio PDX1+ / SOX2+ domain length
0.95
0.90
0.85
0.80
0.75
n.s.
Control E9.5
Bapx1Cre/+;Sufuf/f;Spopf/f
D
E12.5 Control
St
Panc
Int
E
St
Int
F
BV
G
PDX1 SOX9
NKX6.1 SOX9
BrdU NKX6.1
BrdU NGN3
H
J
M
P
E13.5 Control
I
K
N
Q
Control E13.5
Bapx1Cre/+;Sufuf/f;Spopf/f
L
Prop. of NKX6.1+ vs. SOX9+ progenitors
1.0
0.8
0.6
0.4
0.2
0.0
n.s.
O
Prop. of BrdU+ NKX6.1+ progenitors
0.5
0.4
0.3
0.2
0.1
0.0
R
Prop. of BrdU+ NGN3+ progenitors
0.20
0.15
0.10
0.05
0.00

Supplementary Figure 6.

Characterization of *Sufu Spop* mesenchymal mutants during compartmental specification.
A-B) Whole mount immunostaining for the PDX1^{+} pancreatic progenitor pool, observed as buds evaginating from the embryonic gut tube, in E10.5 controls (A) and *Bapx1*$^{Cre/+}$;*Sufu*$^{f/f}$;*Spop*$^{f/f}$ mutants (B). **C)** Quantification of the ratio of PDX1^{+} versus SOX2^{+} domain length along the E9.5 gut tube in controls and *Bapx1*$^{Cre/+}$;*Sufu*$^{f/f}$;*Spop*$^{f/f}$ mutants (n=3 samples each genotype). **D)** E12.5 control embryos exhibit an external pancreas (Panc) located adjacent to the stomach (St) and proximal intestine (Int). **E)** At E12.5, *Bapx1*$^{Cre/+}$;*Sufu*$^{f/f}$;*Spop*$^{f/f}$ embryos already lose an externally visible pancreas and exhibit mesenchymal hyperplasia. **F)** Immunostaining for SMA and SM22 in E12.5 controls demonstrates expression in the stomach mesenchyme and absence from the pancreatic mesenchyme (dashed outline). BV- blood vessel. **G)** In E12.5 *Bapx1*$^{Cre/+}$;*Sufu*$^{f/f}$;*Spop*$^{f/f}$ embryos, mutant pancreatic mesenchyme stains for SMA and SM22 (dashed outline denotes pancreas). **H-I)** SOX9^{+} PDX1^{+} co-localization in the pancreatic progenitors of E13.5 control (H) and *Bapx1*$^{Cre/+}$;*Sufu*$^{f/f}$;*Spop*$^{f/f}$ mutants (I). **J,K)** Compartmentalization of immuno-stained NKX6.1^{+} trunk cells within the SOX9^{+} progenitor population in E13.5 control (J) versus *Bapx1*$^{Cre/+}$;*Sufu*$^{f/f}$;*Spop*$^{f/f}$ mutants (K). **L)** Quantification of the proportion of NKX6.1^{+} cell number as a proportion of total NKX6.1^{+} and/or SOX9^{+} total cell number (n=4 samples per genotype). **M,N)** Immunostaining of NKX6.1^{+} trunk progenitor cells marked by thymidine analog, 5-bromo-2'-deoxyuridine (BrdU) one hour after administration, in E13.5 control (M) versus *Bapx1*$^{Cre/+}$;*Sufu*$^{f/f}$;*Spop*$^{f/f}$ mutants (N). **O)** Quantification of the proportion of NKX6.1^{+} cells co-labelled with BrdU as a measure of trunk endocrine progenitor proliferation in mutants versus controls (n=3 control, 4 mutant samples). **P,Q)** Immunostaining of NGN3^{+} committed endocrine progenitors marked by BrdU one hour after administration, in E13.5 control (P) versus *Bapx1*$^{Cre/+}$;*Sufu*$^{f/f}$;*Spop*$^{f/f}$ mutants (Q). **R)** Quantification of proliferation, as marked by BrdU, in NGN3^{+} endocrine progenitors of E13.5 mutants and controls (n=3 control, 4 mutant samples). Data are means ± SEM. n.s. denotes not significant, * denotes $p<0.05$ by Student's un-paired t-test. Scale bars: Whole mount- 1 mm, Whole mount immunofluorescence- 90 µm, Immunofluorescence- 100 µm.

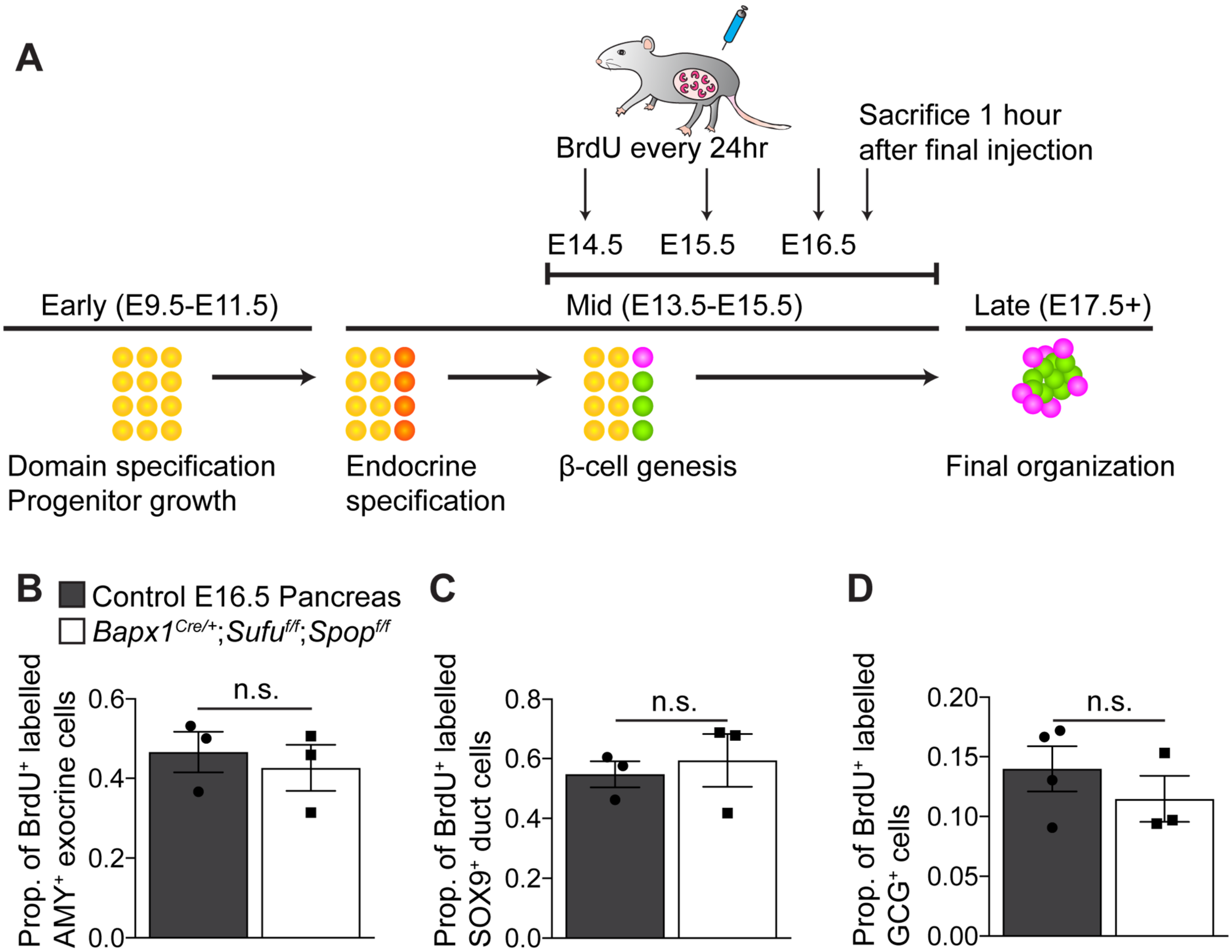

Supplementary Figure 7.

Characterization of cell population growth in *Sufu Spop* mesenchymal mutants via BrdU tracing. **A)** Schematic for beta cell genesis tracing experiments with thymidine analog, 5-bromo-2'-deoxyuridine (BrdU). BrdU was administered every 24 hours from E14.5 to E16.5 via intraperitoneal injection of pregnant dams and harvested 1 hour after the final injection on E16.5. As intrinsic replication of beta cells during this time is rare[1] and beta cell differentiation occurs after E13.5, BrdU uptake from E14.5-E16.5 will predominantly mark newly synthesized endocrine progenitors that then differentiate into endocrine sub-types. Co-staining BrdU with INS will then mark endocrine progenitors that have differentiated into INS^+ beta cells. **B)** Quantification of the proportion of AMY^+ acinar cells in *Bapx1*$^{Cre/+}$;*Sufu*$^{f/f}$;*Spop*$^{f/f}$ mutants marked by BrdU after E14.5-E16.5 tracing as compared to controls (n=3 samples per genotype). **C)** Quantification of the proportion of $SOX9^+$ duct cells marked by BrdU after E14.5-E16.5 tracing in *Bapx1*$^{Cre/+}$;*Sufu*$^{f/f}$;*Spop*$^{f/f}$ mutants compared to controls (n=3 samples per genotype). **D)** Quantification of the proportion of GCG^+ glucagon cells marked by BrdU after E14.5-E16.5 tracing in *Bapx1*$^{Cre/+}$;*Sufu*$^{f/f}$;*Spop*$^{f/f}$ mutants compared to controls (n=3 samples per genotype). Data are means ± SEM. n.s. denotes not significant, * denotes $p<0.05$ by Student's un-paired t-test.

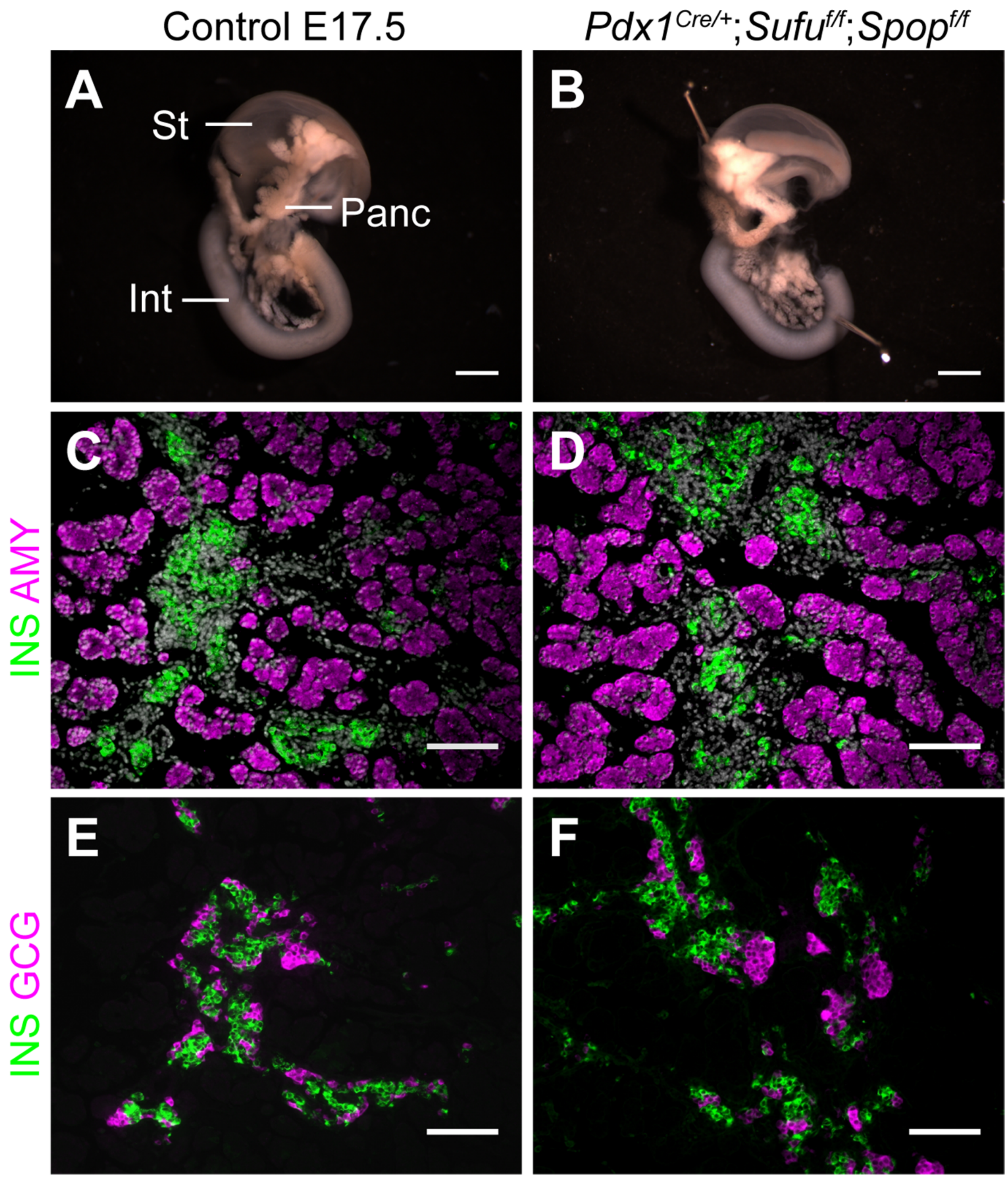

Supplementary Figure 8.

Loss of pancreatic epithelial *Sufu* and *Spop* does not cause discernable developmental defects. **A-B)** *Pdx1*$^{Cre/+}$;*Sufu*$^{f/f}$;*Spop*$^{f/f}$ embryos (B) phenotypically appear normal as compared to E17.5 controls (A). St- stomach, Panc- pancreas, Int- Intestine. **C-D)** Immunostaining for INS and AMY indicates proper differentiation of exocrine and endocrine compartments in E17.5 controls (C) and *Pdx1*$^{Cre/+}$;*Sufu*$^{f/f}$;*Spop*$^{f/f}$ mutants (D). **E-F)** Endocrine makeup of INS$^+$ versus GCG$^+$ cells in *Pdx1*$^{Cre/+}$;*Sufu*$^{f/f}$;*Spop*$^{f/f}$ embryos (F) remains comparable to controls (E). Scale bars: Whole mount- 1 mm, Immunofluorescence- 100 µm.

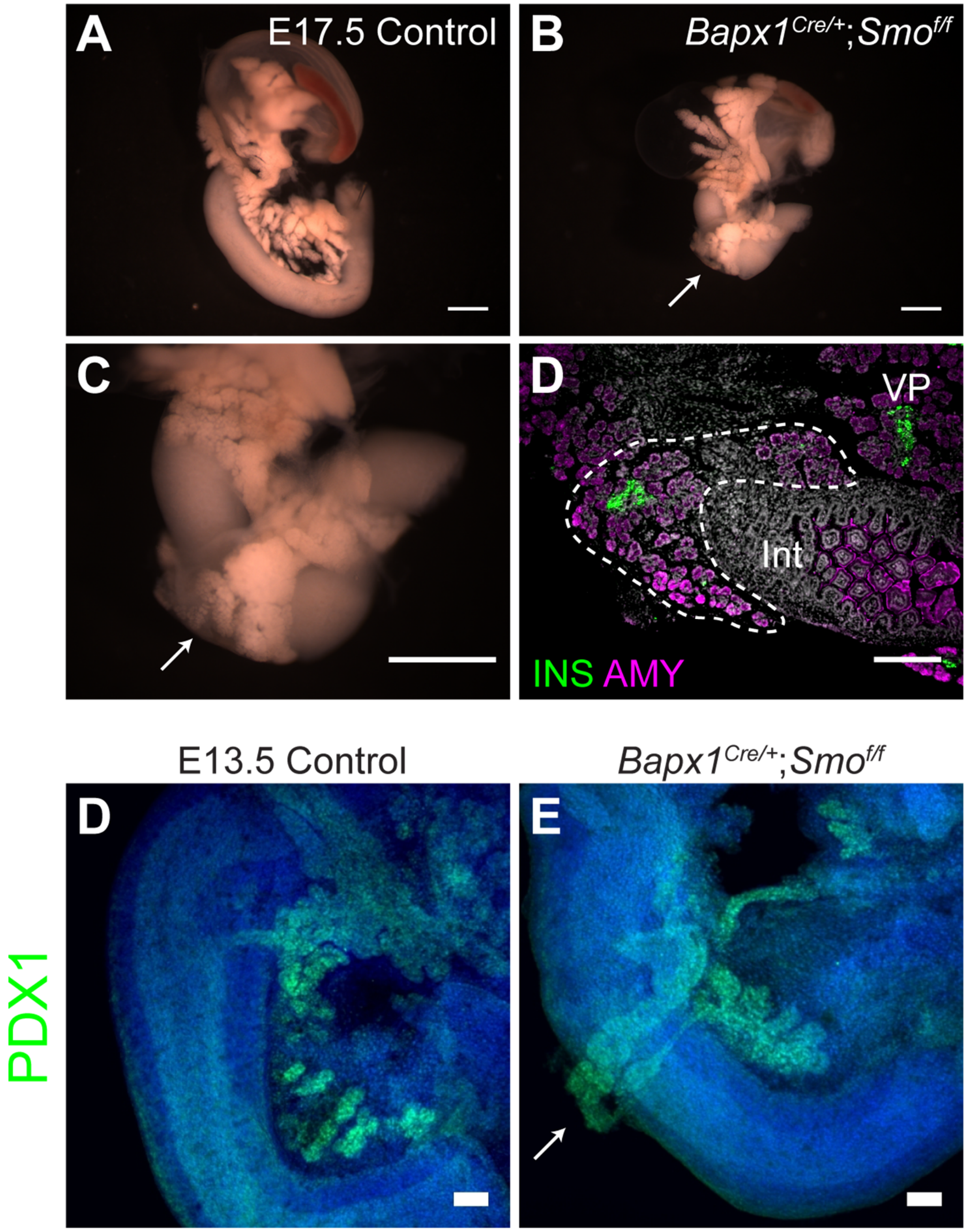

Supplementary Figure 9.

Mesenchymal Hedgehog inhibition leads to ectopic epithelial branching in the pancreas. A) E17.5 control pancreata, exhibit a ventral domain (VP) nestled into the proximal intestinal loop (Int). **B)** Inhibition of mesenchymal Hedgehog signaling in *Bapx1*$^{Cre/+}$;*Smo*$^{f/f}$ embryos leads to ectopic branching (arrow) from the ventral pancreatic domain to form an annulus around the intestine. **C)** Higher magnification image of pancreatic annulus in *Bapx1*$^{Cre/+}$;*Smo*$^{f/f}$ embryos (arrow). **D)** This annulus (dashed outline) stains for INS and AMY, indicative of pancreatic identity. **E-F)** Whole mount immunofluorescence for PDX1 in E13.5 controls (E) versus *Bapx1*$^{Cre/+}$;*Smo*$^{f/f}$ mutants (F) reveals ectopic PDX1^{+} pancreatic branching (arrow) during branching morphogenesis. VP- Ventral pancreas. Int- Intestine. Scale bars: Whole mount- 1 mm, Immunofluorescence- 100 µm.

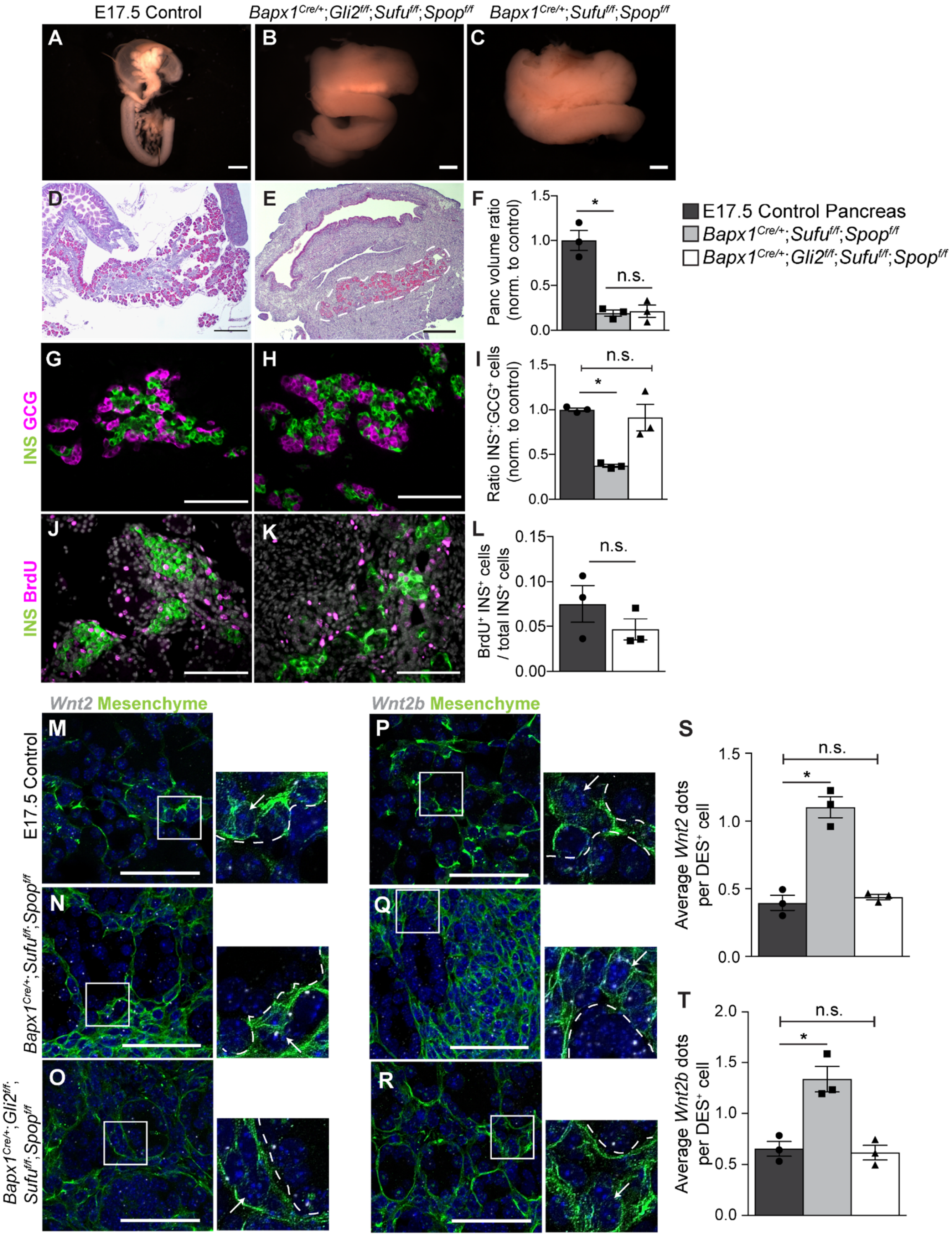

E17.5 Control
Bapx1Cre/+;Gli2f/f;Sufuf/f;Spopf/f
Bapx1Cre/+;Sufuf/f;Spopf/f
A
B
C
D
E
F
Panc volume ratio (norm. to control)
*
n.s.
E17.5 Control Pancreas
Bapx1Cre/+;Sufuf/f;Spopf/f
Bapx1Cre/+;Gli2f/f;Sufuf/f;Spopf/f
G
H
INS GCG
I
Ratio INS+:GCG+ cells (norm. to control)
J
K
INS BrdU
L
BrdU+ INS+ cells / total INS+ cells
Wnt2 Mesenchyme
Wnt2b Mesenchyme
M
N
O
P
Q
R
E17.5 Control
S
Average Wnt2 dots per DES+ cell
T
Average Wnt2b dots per DES+ cell

Supplementary Figure 10.

Mesenchymal deletion of *Gli2* partially rescues *Sufu Spop* mesenchymal mutant defects.
A-C) E17.5 gut of embryos harboring *Gli2* deletion in mesenchymal *Sufu* and *Spop* mutant background ($Bapx1^{Cre/+}$;$Gli2^{f/f}$;$Sufu^{f/f}$;$Spop^{f/f}$) (B) as compared to E17.5 control (A) and the original $Bapx1^{Cre/+}$;$Sufu^{f/f}$;$Spop^{f/f}$ mutant (C). **D)** Histological staining of E17.5 control demonstrating predominantly cytoplasmic makeup of pancreatic tissue (pink eosin). **E)** Histological staining of E17.5 $Bapx1^{Cre/+}$;$Gli2^{f/f}$;$Sufu^{f/f}$;$Spop^{f/f}$ guts revealing integration of cytoplasmic pancreatic tissue (dashed outline denotes pancreas). **F)** Comparison of the ratios in E17.5 pancreatic volume between $Bapx1^{Cre/+}$;$Sufu^{f/f}$;$Spop^{f/f}$ mutants and their controls versus $Bapx1^{Cre/+}$;$Gli2^{f/f}$;$Sufu^{f/f}$;$Spop^{f/f}$ rescues and their controls (n=3 samples of each genotype). **G-H)** Immunostaining for INS and GCG in E17.5 control (G) and $Bapx1^{Cre/+}$;$Gli2^{f/f}$;$Sufu^{f/f}$;$Spop^{f/f}$ embryos (H). **I)** Comparison of INS^+:GCG^+ cell ratios between $Bapx1^{Cre/+}$;$Sufu^{f/f}$;$Spop^{f/f}$ mutants and their controls versus $Bapx1^{Cre/+}$;$Gli2^{f/f}$;$Sufu^{f/f}$;$Spop^{f/f}$ rescues and their controls (n=3 samples of each genotype). **J-K)** Immunostaining for INS and proliferation marker, 5-bromo-2'-deoxyuridine (BrdU) in E17.5 control (J) and $Bapx1^{Cre/+}$;$Gli2^{f/f}$;$Sufu^{f/f}$;$Spop^{f/f}$ embryos (K). **L)** Assessment of INS^+ beta cell proliferation in E17.5 $Bapx1^{Cre/+}$;$Gli2^{f/f}$;$Sufu^{f/f}$;$Spop^{f/f}$ versus control embryos injected with BrdU one hour prior to sacrifice (n=3 samples per genotype). **M-O)** Representative single molecule fluorescent *in situ* hybridization (smFISH) images for Wnt ligand, *Wnt2*, in E17.5 control (M), $Bapx1^{Cre/+}$;$Sufu^{f/f}$;$Spop^{f/f}$ (N), and $Bapx1^{Cre/+}$;$Gli2^{f/f}$;$Sufu^{f/f}$;$Spop^{f/f}$ (O) pancreata co-stained with DES to mark mesenchymal cells. **P-R)** Representative smFISH images for Wnt ligand, *Wnt2b*, in E17.5 control (P), $Bapx1^{Cre/+}$;$Sufu^{f/f}$;$Spop^{f/f}$ (Q), and $Bapx1^{Cre/+}$;$Gli2^{f/f}$;$Sufu^{f/f}$;$Spop^{f/f}$ (R) pancreata co-stained with DES. **S-T)** Quantification of smFISH dots per DES^+ mesenchymal cell for Wnt ligands, *Wnt2* (S) and *Wnt2b* (T) of E17.5 $Bapx1^{Cre/+}$;$Gli2^{f/f}$;$Sufu^{f/f}$;$Spop^{f/f}$ embryos versus controls and $Bapx1^{Cre/+}$;$Sufu^{f/f}$;$Spop^{f/f}$ mutants (n=3 samples per genotype). White boxes correspond to zoomed-in panels. Dashed line separates DES^- epithelium and DES^+ mesenchyme. Arrows indicate representative mesenchymal cells. Data are means ± SEM. n.s. denotes not significant, * denotes $p<0.05$ by Student's un-paired t-test. Scale bars: Whole mount- 1 mm, Histology- 500 µm, Immunofluorescence- 100 µm, smFISH- 50 µm.

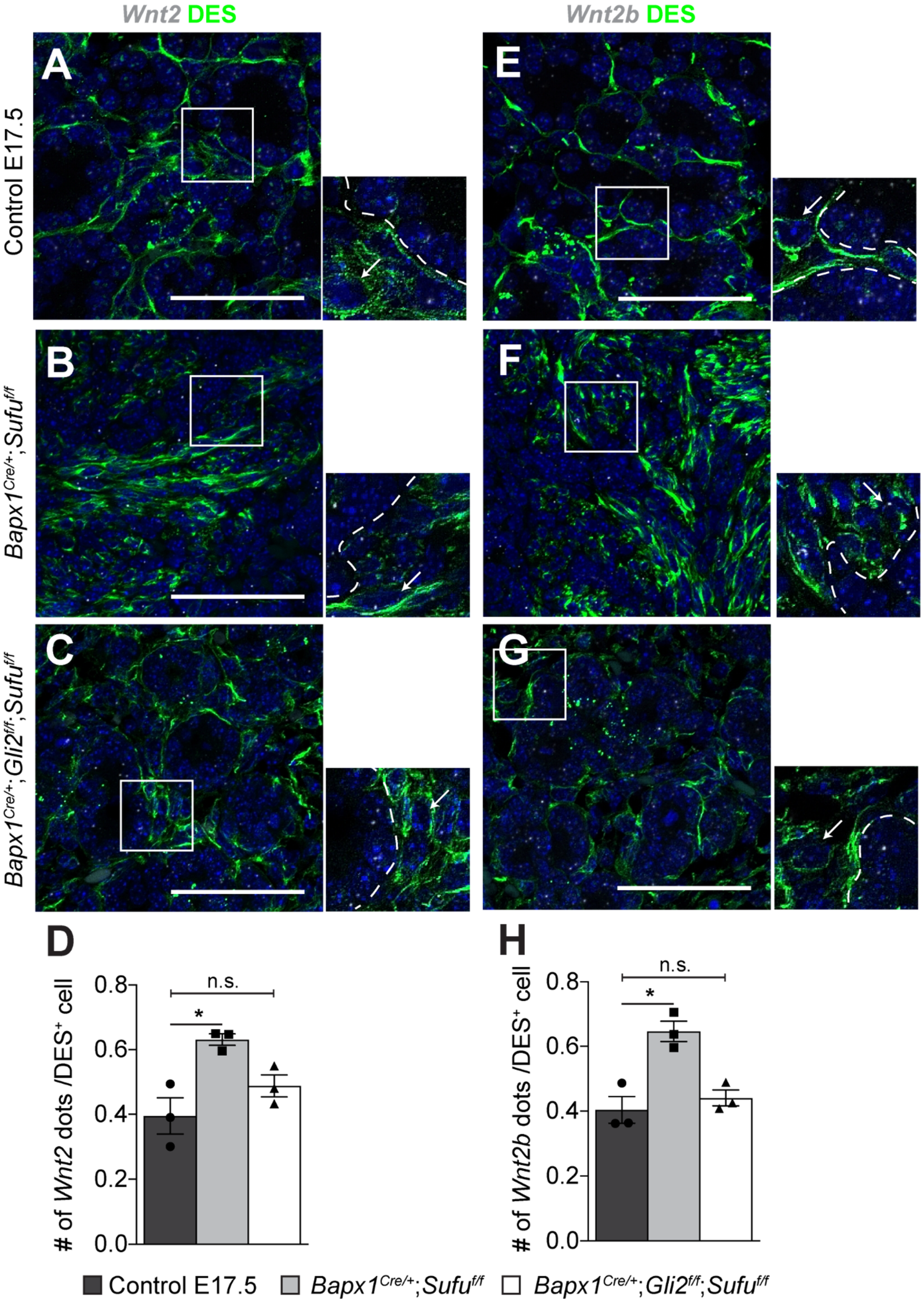
Wnt2 DES
Wnt2b DES
A
B
C
D
E
F
G
H
Control E17.5
Bapx1Cre/+;Sufuf/f
Bapx1Cre/+;Gli2f/f;Sufuf/f
# of Wnt2 dots /DES+ cell
# of Wnt2b dots /DES+ cell
0.0
0.2
0.4
0.6
0.8
n.s.
*
Control E17.5
Bapx1Cre/+;Sufuf/f
Bapx1Cre/+;Gli2f/f;Sufuf/f

Supplementary Figure 11.

Mesenchymal deletion of *Gli2* recovers Wnt ligand overexpression in *Bapx1*$^{Cre/+}$;*Sufu*$^{f/f}$ mutants. **A-C)** Representative single molecule fluorescent *in situ* hybridization (smFISH) images for Wnt ligand, *Wnt2*, in E17.5 control (A), *Bapx1*$^{Cre/+}$;*Sufu*$^{f/f}$ (B), and *Bapx1*$^{Cre/+}$;*Gli2*$^{f/f}$;*Sufu*$^{f/f}$ (C) pancreata co-stained with DES to mark mesenchymal cells. **D)** Quantification of smFISH dots per DES^{+} mesenchymal cell for Wnt ligand, *Wnt2*, in E17.5 control, *Bapx1*$^{Cre/+}$;*Sufu*$^{f/f}$, and *Bapx1*$^{Cre/+}$;*Gli2*$^{f/f}$;*Sufu*$^{f/f}$ embryos (n=3 samples per genotype). **E-G)** Representative smFISH images for Wnt ligand, *Wnt2b*, in E17.5 control (E), *Bapx1*$^{Cre/+}$;*Sufu*$^{f/f}$ (F), and *Bapx1*$^{Cre/+}$;*Gli2*$^{f/f}$;*Sufu*$^{f/f}$ (G) pancreata co-stained with DES. **H)** Quantification of smFISH dots per DES^{+} mesenchymal cell for Wnt ligand, *Wnt2b*, in E17.5 control, *Bapx1*$^{Cre/+}$;*Sufu*$^{f/f}$, and *Bapx1*$^{Cre/+}$;*Gli2*$^{f/f}$;*Sufu*$^{f/f}$ embryos (n=3 samples per genotype). White boxes correspond to zoomed-in panels. Dashed line separates DES^{-} epithelium and DES^{+} mesenchyme. Arrows indicate representative mesenchymal cells. Data are means ± SEM. n.s. denotes not significant, * denotes $p<0.05$ by Student's un-paired t-test. Scale bars: 50 µm.

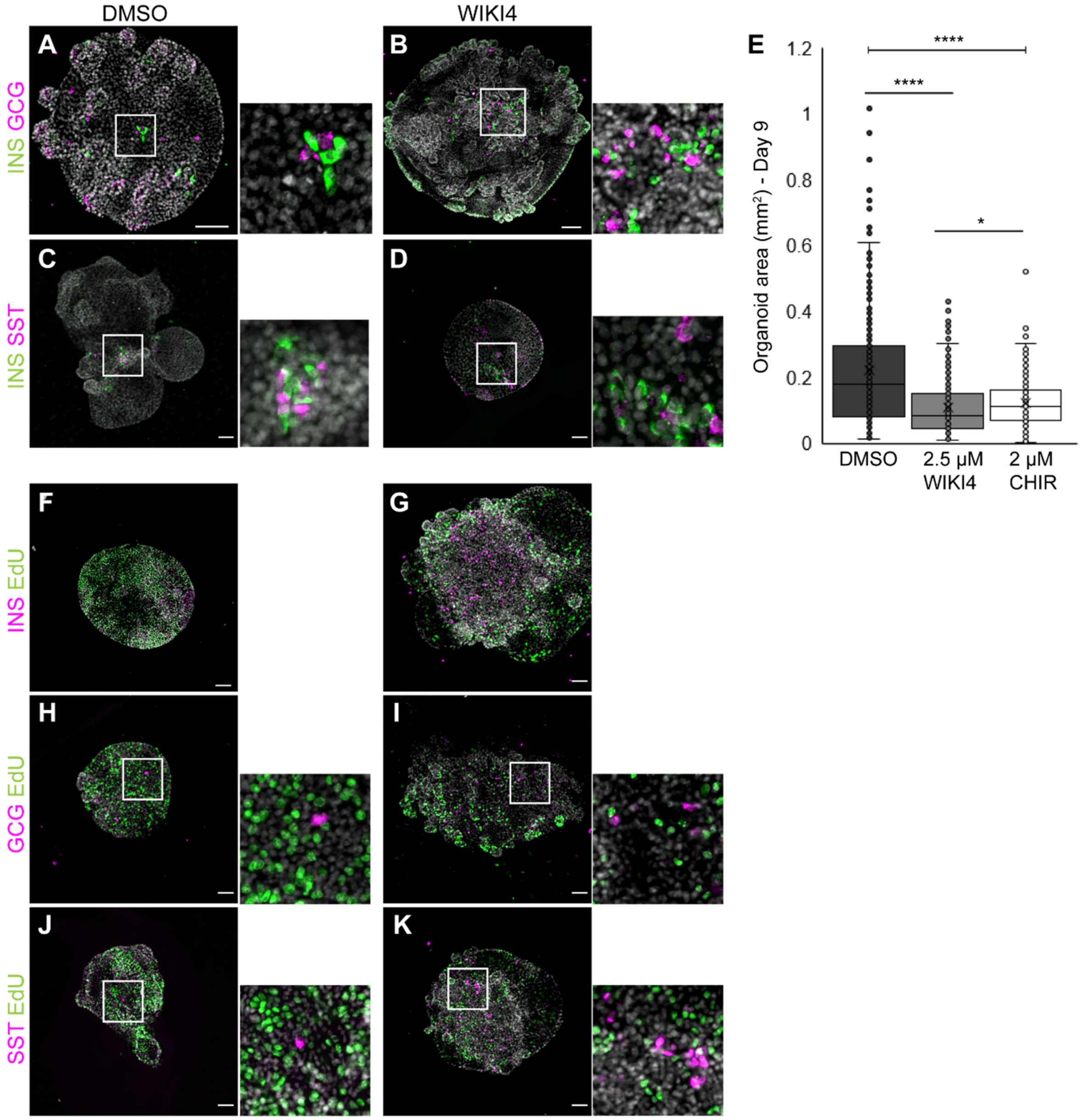

Supplementary Figure 12.

Characterization of pancreatic epithelial organoids derived from in vivo pancreatic progenitors. **A-B)** Representative immunostaining for GCG with INS in DMSO- (A) and WIKI4-treated organoids (B) at day 9 of culture. **C-D)** Representative immunostaining for SST with INS in DMSO- (C) and WIKI4-treated organoids (D) at day 9 of culture. **E)** Comparison of organoid area of DMSO (n=294), WIKI4 (n=326), and CHIR (n=504) treated organoids at day 9 of culture. Mid line of box denotes median, upper line denotes third quartile, lower line denotes first quartile. Whiskers indicate maximum (top) and minimum (bottom). * denotes $p<0.05$, **** denotes $p<0.00005$ by Student's un-paired t-test. **F-K)** Representative images of day 9 DMSO and WIKI4-treated organoids treated with 5-Chloro-2'-deoxyuridine (EdU) for 1 hour before harvest and co-stained with INS (F-G), GCG (H-I), and SST (J-K) to assess endocrine cell proliferation. White boxes correspond to zoomed-in panels. Scale bars: 100 µm.

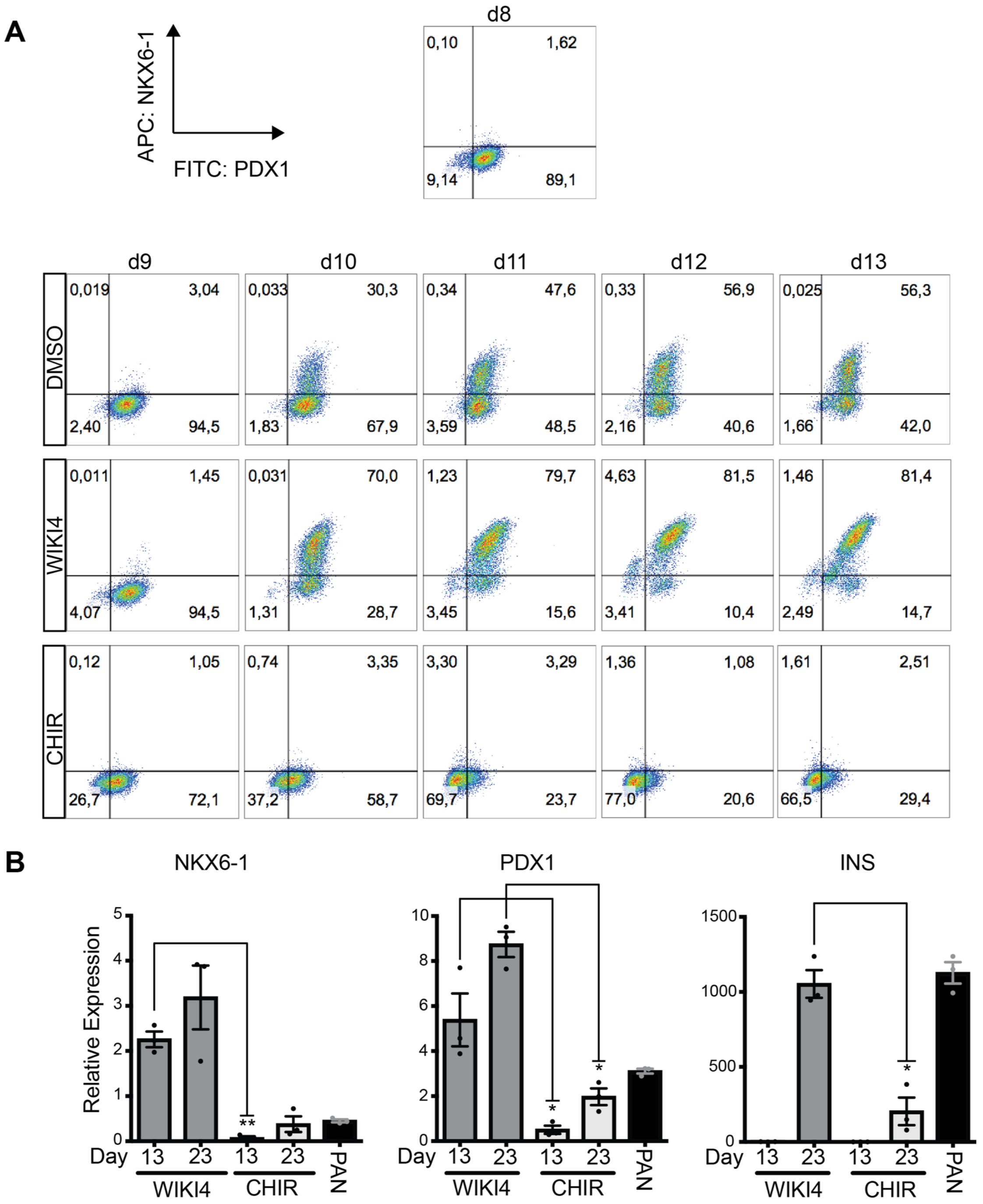

Supplementary Figure 13.

Modulation of WNT signaling during human stem cell-derived pancreatic differentiation. A) Representative flow cytometry plots from day 8 to day 13 of cultures differentiated with DMSO, WIKI4 or CHIR at stage 4. Cells were stained with anti-PDX1 and anti-NKX6-1. **B)** qPCR analysis of *NKX6-1, PDX1* and *INS* at days 13 and 23 of differentiation in the presence of WIKI4 or CHIR (n=3 independent experiments). Relative expression levels are normalized to housekeeping gene *TATA-binding protein* (*TBP*) and compared to adult pancreas. Data are means ± SEM. * denotes p<0.05, ** denotes p<0.01 by Student's paired t-test.

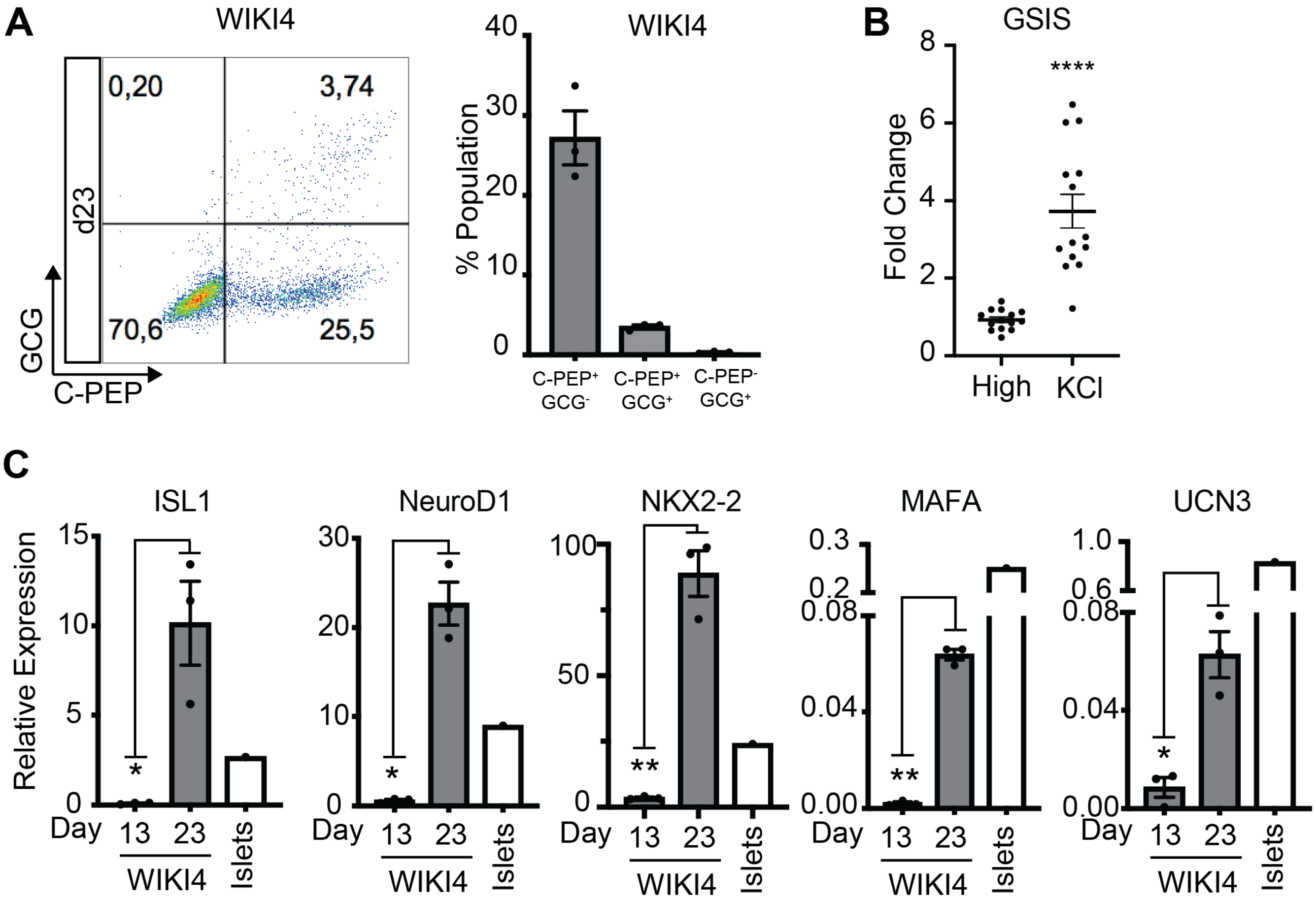

Supplementary Figure 14.

Characterization of WIKI4-derived beta-like cell populations. A) Representative flow cytometry plot of day 23 cultures differentiated with WIKI4 at stage 4. Cells were stained with anti-C-Peptide (C-PEP) and anti-Glucagon (GCG). Quantification of the percentage of C-PEP$^+$/GCG$^-$, C-PEP$^+$/GCG$^+$ and C-PEP$^-$/GCG$^+$ endocrine cells generated at day 23 of differentiation after treatment with WIKI4 (n=4 independent experiments). **B)** Glucose stimulated insulin secretion (GSIS) assay was performed at day 35/36 of differentiation. Data are presented as fold changes of insulin secreted after 16.7 mM glucose (high) and 25 mM KCl stimulation compared to secretion at 2.8 mM glucose (n=3 biological replicates, with 4-5 technical replicates). Data are means ± SEM. **** denotes p<0.0001 by Student's paired t-test. **C)** Gene expression analyses of endocrine and beta cell markers (*ISL1, NeuroD1, NKX2-2, MAFA* and *UCN3*) in WIKI4-derived cells on day 13 and day 23 of differentiation (n=3 independent experiments). Relative expression levels are normalized to housekeeping gene *TATA-binding protein* (*TBP*) and compared to adult human islets. Data are means ± SEM. * denotes p<0.05, ** denotes p<0.01 by Student's paired t-test.

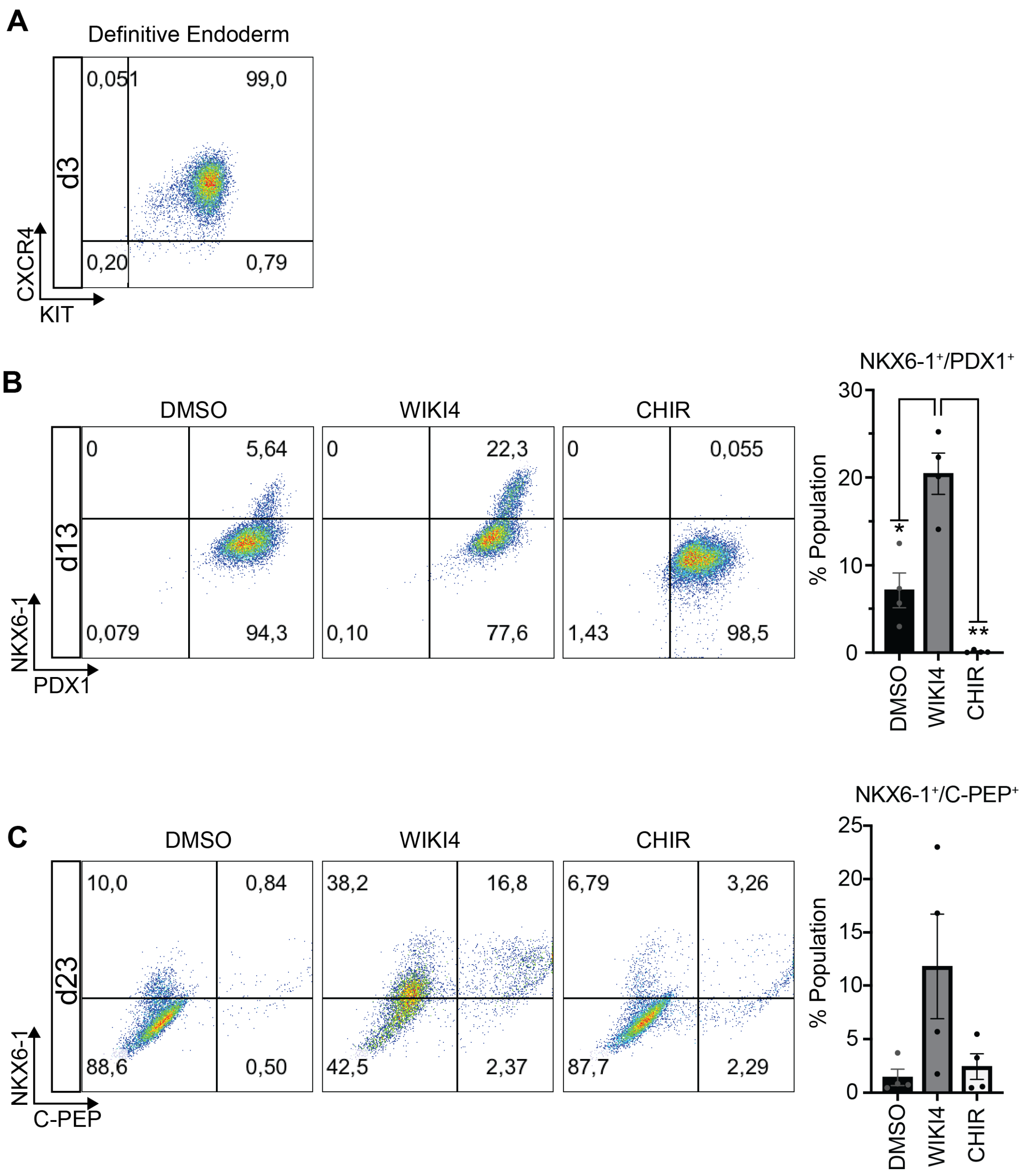

Supplementary Figure 15.

The effect of WNT inhibition in stem cell-derived pancreatic endoderm is not cell line specific. A human induced pluripotent stem cell line, BJ-iPSC-1, was differentiated to pancreatic beta-like cells according to Figure 7A. **A)** Flow cytometry profile of CXCR4 and c-KIT to demonstrate efficient generation of definitive endoderm at day 3. **B)** Representative flow cytometry plot and quantification of the percentage of NKX6-1$^+$/PDX1$^+$ cells generated at day 13 of differentiation, after treatment with DMSO, WIKI4 or CHIR at stage 4 of differentiation (n=4 independent experiments). Data are means ± SEM. * denotes $p<0.05$, ** denotes $p<0.01$ by one-way ANOVA. **C)** Representative flow cytometry plot and quantification of the percentage of NKX6-1$^+$/C-PEP$^+$ beta-like cells generated at day 23 of differentiation, after treatment with DMSO, WIKI4 or CHIR at stage 4 of differentiation (n=4 independent experiments). Data are means ± SEM. * denotes $p<0.05$, ** denotes $p<0.01$ by one-way ANOVA.

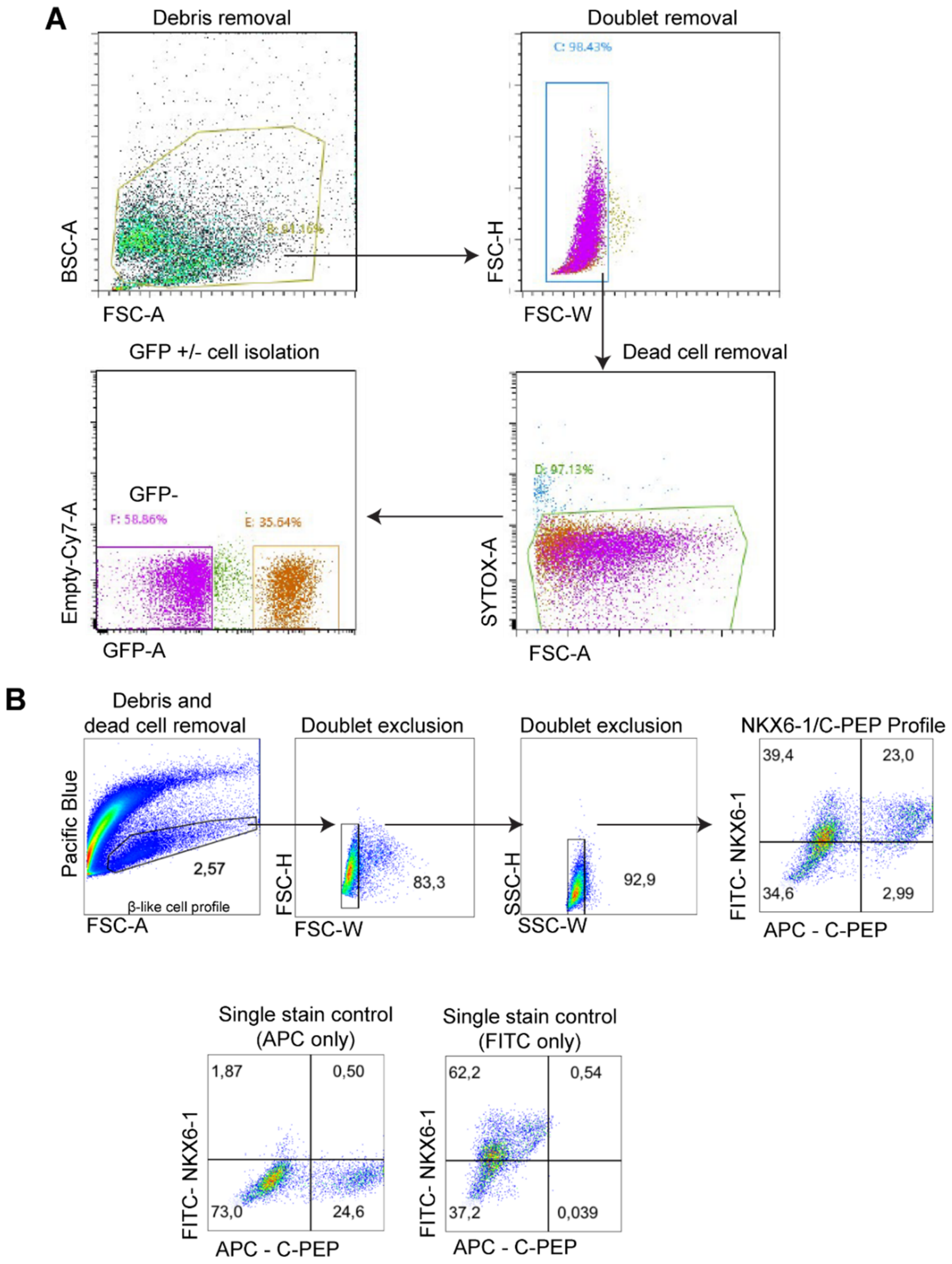

Supplementary Figure 16.

Gating strategies used for cell sorting. A) Gating strategy to sort GFP^{+} mesenchyme and GFP^{-} non-mesenchyme from *Bapx1*$^{Cre/+}$;*ROSA26*$^{mT/mG}$ and *Bapx1*$^{Cre/+}$;*ROSA26*$^{mT/mG}$;*Sufu*$^{f/f}$;*Spop*$^{f/f}$ embryonic guts for RNA-seq analysis in Fig. 1 and 5, qPCR in Fig. 2E and Supplementary Fig. 2E, and the FACs plots in Supplementary Fig. 1A and 3C,D. **B)** For hPSC studies, debris and dead cells were removed using zombie violet viability dye (Pacific Blue) versus FSC-A. Doublets were excluded using FSC-H versus FSC-W and then SSC-H versus SSC-W (Upper panels). Flow gate was set based on single stain (Lower panels). hPSC gating strategy was used to generate data in Fig. 7B-F, and Supplementary Fig. 13-15.

Supplementary Reference

1. Sander, M. *et al.* Homeobox gene Nkx6.1 lies downstream of Nkx2.2 in the major pathway of beta-cell formation in the pancreas. *Development* **127**, 5533–40 (2000).
