# Supplementary material for: Sufu- and Spop-mediated downregulation of Hedgehog signaling promotes beta cell differentiation through organ-specific niche signals
Source: Nat Commun. 2019 Oct 11;10:4647. doi: 10.1038/s41467-019-12624-5 (PMC6789033; doi:10.1038/s41467-019-12624-5)
Supplement: Supplementary file 2 — Reporting Summary [file 41467_2019_12624_MOESM2_ESM.pdf]

nature research

Corresponding author(s): Tae-Hee Kim

Last updated by author(s): Aug 25, 2019

Reporting Summary

Nature Research wishes to improve the reproducibility of the work that we publish. This form provides structure for consistency and transparency in reporting. For further information on Nature Research policies, see Authors & Referees and the Editorial Policy Checklist.

Statistics

For all statistical analyses, confirm that the following items are present in the figure legend, table legend, main text, or Methods section.

n/a	Confirmed	
☐	☒	The exact sample size (n) for each experimental group/condition, given as a discrete number and unit of measurement
☐	☒	A statement on whether measurements were taken from distinct samples or whether the same sample was measured repeatedly
☐	☒	The statistical test(s) used AND whether they are one- or two-sided<br>*Only common tests should be described solely by name; describe more complex techniques in the Methods section.*
☒	☐	A description of all covariates tested
☒	☐	A description of any assumptions or corrections, such as tests of normality and adjustment for multiple comparisons
☐	☒	A full description of the statistical parameters including central tendency (e.g. means) or other basic estimates (e.g. regression coefficient) AND variation (e.g. standard deviation) or associated estimates of uncertainty (e.g. confidence intervals)
☐	☒	For null hypothesis testing, the test statistic (e.g. F, t, r) with confidence intervals, effect sizes, degrees of freedom and P value noted<br>*Give P values as exact values whenever suitable.*
☒	☐	For Bayesian analysis, information on the choice of priors and Markov chain Monte Carlo settings
☒	☐	For hierarchical and complex designs, identification of the appropriate level for tests and full reporting of outcomes
☒	☐	Estimates of effect sizes (e.g. Cohen's d, Pearson's r), indicating how they were calculated

Our web collection on statistics for biologists contains articles on many of the points above.

Software and code

Policy information about availability of computer code

Data collection	No software was used for data collection
Data analysis	Read trimming: trimmomatic v0.32<br>Genome alignment: STAR v2.5.1b<br>Gene-level counting: featureCounts v1.5.0<br>Differential expression analysis: DESeq2 v1.14.1<br>GO Enrichment: gProfiler rev 1741 build date 2017-10-19<br>Enrichment Map of GO terms: cytoscape v3.6.0 using the EnrichmentMap app v2.0.1<br>A custom R/Sweave script integrating the above analysis will be submitted with example data.<br>ImageJ for image analysis

For manuscripts utilizing custom algorithms or software that are central to the research but not yet described in published literature, software must be made available to editors/reviewers. We strongly encourage code deposition in a community repository (e.g. GitHub). See the Nature Research guidelines for submitting code & software for further information.

Data

Policy information about availability of data

All manuscripts must include a data availability statement. This statement should provide the following information, where applicable:

- Accession codes, unique identifiers, or web links for publicly available datasets
- A list of figures that have associated raw data
- A description of any restrictions on data availability

RNA-seq data generated in this study have been deposited in GEO under the accession GSE123136.
Raw fastq data is available in SRA under the accession SRP171166.
Source data underlying Figure 1b-d, 5b, and Supplementary Figure 1b-d are included as a Source Data File.

Target gene lists for BMP signaling were obtained from doi: 10.1101/gr.092114.109 (Supplementary Tables 3+4)
All other relevant data supporting the key findings of this study are available within the article, in the supplementary files, or from the corresponding author upon reasonable request.

Field-specific reporting

Please select the one below that is the best fit for your research. If you are not sure, read the appropriate sections before making your selection.

☒ Life sciences ☐ Behavioural & social sciences ☐ Ecological, evolutionary & environmental sciences

For a reference copy of the document with all sections, see nature.com/documents/nr-reporting-summary-flat.pdf

Life sciences study design

All studies must disclose on these points even when the disclosure is negative.

Sample size	Sample size was dependent on litter sizes produced from genetic crosses. A minimum sample number of 3 was used for all analyses of genetic mouse models.
Data exclusions	No data was excluded
Replication	Bulk mRNA-seq for E13.5 stomach, pancreas, intestine, and mutants: 2 replicates each. PCA analysis demonstrated strong clustering between replicates.
Randomization	This study does not contain groups that require randomization.
Blinding	Samples were analyzed in random order. However, due to the dramatic phenotypes observed in our mutants, complete blinding was not always possible.

Reporting for specific materials, systems and methods

We require information from authors about some types of materials, experimental systems and methods used in many studies. Here, indicate whether each material, system or method listed is relevant to your study. If you are not sure if a list item applies to your research, read the appropriate section before selecting a response.

Materials & experimental systems

n/a	Involved in the study
☐	☒ Antibodies
☐	☒ Eukaryotic cell lines
☒	☐ Palaeontology
☐	☒ Animals and other organisms
☒	☐ Human research participants
☒	☐ Clinical data

Methods

n/a	Involved in the study
☒	☐ ChIP-seq
☐	☒ Flow cytometry
☒	☐ MRI-based neuroimaging

Antibodies

Antibodies used	mouse anti-insulin (Sigma, I2018), rabbit anti-insulin (Abcam, ab181547), rabbit anti-amylase (Abcam, ab21156), mouse anti-glucagon (Abcam, ab10988),mouse anti-smooth muscle actin (Sigma, A2547), rabbit anti-desmin (Abcam, ab32362), rabbit anti-SM22 (Abcam, ab14106), mouse anti-PDX1 (DSHB, F109-D12), rabbit anti-SOX2 (Abcam, ab97959), rat anti-BrDU (Abcam, 6326), rabbit anti-SOX9 (EMD Millipore, AB5535), mouse anti-NGN3 (DSHB, F25A1B3), mouse anti-NKX6.1 (DSHB, F55A12), rat anti-somatostatin [M09204] (Abcam, ab30788), rat anti-ghrelin (R&D systems, MAB8200), mouse anti-EpCam [G8.8] (DSHB, G8.8). Secondary antibodies: AlexaFlour488 and AlexFlour568.
Validation	mouse anti-insulin (Sigma, I2018), rabbit anti-insulin (Abcam, ab181547), rabbit anti-amylase (Abcam, ab21156), mouse anti-glucagon (Abcam, ab10988),mouse anti-smooth muscle actin (Sigma, A2547), rabbit anti-desmin (Abcam, ab32362), rabbit anti-SM22 (Abcam, ab14106), rabbit anti-SOX2 (Abcam, ab97959), rat anti-BrDU (Abcam, 6326), rabbit anti-SOX9 (EMD Millipore, AB5535), rat anti-somatostatin [M09204] (Abcam, ab30788), rat anti-ghrelin (R&D systems, MAB8200) were all validated by the supplier.<br>For mouse anti-PDX1 (DSHB, F109-D12), mouse anti-NGN3 (DSHB, F25A1B3), mouse anti-NKX6.1 (DSHB, F55A12), mouse anti-EpCam [G8.8] (DSHB, G8.8) from DSHB, see company website for originating publication of the antibody.<br>All antibodies were tested on control pancreatic tissue prior to use for experiments.

Eukaryotic cell lines

Policy information about cell lines

Cell line source(s)	H1 embryonic stem cell - WiCell Research Institute (Madison WI, USA); BJ-iPSC-1 induced pluripotent stem cell line - Drs. Toshi Araki and Ben Neel (New York University, NY, USA). Dr. Nostro has approval from the Stem Cell Oversight Committee (Canadian Institute of Health Research) to conduct work with SCOC-approved H1 and human iPSCs
Authentication	Cell lines were karyotyped upon receipt to check for the presence of abnormal chromosomes
Mycoplasma contamination	Cell lines were tested and negative for mycoplasma contamination
Commonly misidentified lines (See ICLAC register)	No commonly misidentified lines used in this study.

Animals and other organisms

Policy information about studies involving animals; ARRIVE guidelines recommended for reporting animal research

Laboratory animals	Bapx1Cre, ROSA26(mT/mG), Pdx1Cre, Sufuf/f, Spopf/f, Gli2f/f, Smof/f, and CD-1 mice
Wild animals	Our study did not involve wild animals.
Field-collected samples	Our study did not involve samples collected from the field.
Ethics oversight	All procedures involving animals were performed in compliance with the Animals for Research Act of Ontario and the Guidelines of the Canadian Council on Animal Care. The Toronto Centre for Phenogenomics (TCP) Animal Care Committee reviewed and approved all procedures conducted on animals at TCP

Note that full information on the approval of the study protocol must also be provided in the manuscript.

Flow Cytometry

Plots

Confirm that:

☒ The axis labels state the marker and fluorochrome used (e.g. CD4-FITC).

☒ The axis scales are clearly visible. Include numbers along axes only for bottom left plot of group (a 'group' is an analysis of identical markers).

☒ All plots are contour plots with outliers or pseudocolor plots.

☒ A numerical value for number of cells or percentage (with statistics) is provided.

Methodology

Sample preparation	Murine embryonic gut tissue: Trypsin digestion at 37C, manual homogenization, stained with SYTOX Blue Dead Cell Stain, filtered prior to sorting.
Instrument	Beckman Coulter Mo Flo XDP, Mo Flo Astrios sorter, or Sony SH800S Cell Sorter
Software	FlowJo (version 9.8.3)
Cell population abundance	GFP+ mesenchymal cells at E15.5: 40% of live cells
Gating strategy	Debris excluded by FSC-A vs. BSC-A. Doublets excluded by FSC-W vs. FSC-H. Live cells were gated using SYTOX Blue Dead Cell Stain. GFP+/- was determined using GFP negative embryo or unstained controls from the same batch of samples.

☒ Tick this box to confirm that a figure exemplifying the gating strategy is provided in the Supplementary Information.
