# Supplementary material for: Sufu- and Spop-mediated downregulation of Hedgehog signaling promotes beta cell differentiation through organ-specific niche signals
Source: Nat Commun. 2019 Oct 11;10:4647. doi: 10.1038/s41467-019-12624-5 (PMC6789033; doi:10.1038/s41467-019-12624-5)
Supplement: Supplementary file 3 — Description of Additional Supplementary Files [file 41467_2019_12624_MOESM3_ESM.pdf]

Description of Additional Supplementary Files

Supplementary Data 1: Raw read counts for RNA-seq analysis of fluorescence-activated cell sorted E13.5 intestine, stomach, pancreas, and *Bapx1*$^{Cre/+}$;*ROSA26*$^{mT/mG}$;*Sufu*$^{f/f}$;*Spop*$^{f/f}$ mutant pancreas mesenchyme.

Supplementary Data 2: FPKM values derived from RNA-seq raw read counts of fluorescence-activated cell sorted E13.5 intestine, stomach, pancreas, and *Bapx1*$^{Cre/+}$;*ROSA26*$^{mT/mG}$;*Sufu*$^{f/f}$;*Spop*$^{f/f}$ mutant pancreas mesenchyme.

Supplementary Data 3: DESeq2 results table for differential gene analyses between intestine, stomach, and pancreas tissue pairs as well as control versus E13.5 *Bapx1*$^{Cre/+}$;*ROSA26*$^{mT/mG}$;*Sufu*$^{f/f}$;*Spop*$^{f/f}$ pancreatic mesenchyme. Sheets described as follows:
panc_vs_int_deTab: Differential comparison between control pancreatic and intestinal mesenchyme. Positive fold change values indicate relative enrichment in pancreas. Negative values indicate relative enrichment in intestine.
panc_vs_st_deTab: Differential comparison between control pancreatic and stomach mesenchyme. Positive fold change values indicate relative enrichment in pancreas. Negative values indicate relative enrichment in stomach.
st_vs_int_deTab: Differential comparison between control stomach and intestinal mesenchyme. Positive fold change values indicate relative enrichment in stomach. Negative values indicate relative enrichment in intestine.
panc_vs_pancMut_deTab: Differential comparison between E13.5 control versus *Bapx1*$^{Cre/+}$;*ROSA26*$^{mT/mG}$;*Sufu*$^{f/f}$;*Spop*$^{f/f}$ pancreatic mesenchyme. Positive fold change values indicate relative enrichment in control pancreatic mesenchyme. Negative values indicate relative enrichment in mutant pancreatic mesenchyme.

Supplementary Data 4: gProfilerR output of GO enrichment analysis in Generic Enrichment Map (GEM) format, used as input for generating enrichment maps in Fig. 1D.
